# Integrated transcriptomic and metabolomic analyses of a wax deficient citrus mutant exhibiting jasmonic acid-mediated defense against fungal pathogens

**DOI:** 10.1038/s41438-018-0051-0

**Published:** 2018-08-01

**Authors:** Yizhong He, Jingwen Han, Runsheng Liu, Yuduan Ding, Jinqiu Wang, Li Sun, Xiaoming Yang, Yunliu Zeng, Weiwei Wen, Juan Xu, Hongming Zhang, Xiang Yan, Zhaoxing Chen, Zuliang Gu, Hong Chen, Huanqing Tang, Xiuxin Deng, Yunjiang Cheng

**Affiliations:** 1 Key Laboratory of Horticultural Plant Biology, Ministry of Education, Huazhong Agricultural University, Wuhan, 430070 China; 2 Institute of Citrus Science Research of Ganzhou, Ganzhou, 341000 Jiangxi Province China; 3 Research Center of Navel Orange Planting Technology of Anyuan County, Ganzhou, 341000 Jiangxi Province China

## Abstract

Naturally, resistant crop germplasms are important resources for managing the issues of agricultural product safety and environment deterioration. We found a spontaneous mutant of ‘Newhall’ navel orange (*Citrus sinensis* Osbeck) (MT) with broad-spectrum protections against fungal pathogens in the orchard, postharvest-storage, and artificial inoculation conditions. To understand the defense mechanism of MT fruit, we constructed a genome-scale metabolic network that integrated metabolome and transcriptome datasets. The coordinated transcriptomic and metabolic data were enriched in two sub-networks, showing the decrease in very long chain fatty acid (by 41.53%) and cuticular wax synthesis (by 81.34%), and increase in the synthesis of jasmonic acid (JA) (by 95.23%) and JA-induced metabolites such as 5-dimethylnobietin (by 28.37%) in MT. Furthermore, cytological and biochemical analyses confirmed that the response to fungal infection in MT was independent of wax deficiency and was correlated with the levels of jasmonates, and the expression of plant defensin gene *PDF1.2*. Results of exogenous application of MeJA and JA inhibitors such as propyl gallate proved that JA-mediated defense contributes to the strong tolerance against pathogens in MT. Our results indicated that jasmonate biosynthesis and signaling are stimulated by the fatty acid redirection of MT, and participate in the tolerance of pathogenic fungi.

## Orange: Hormone protects mutant orange from fungal pathogens

Elevated hormone signaling underpins fungal protection in a naturally occurring variety of orange found in China. Yunjiang Cheng from Huazhong Agricultural  University in Wuhan, China, and coworkers analyzed all the genes expressed and metabolites produced by a mutant variety of ‘Newhall’ navel orange (*Citrus sinensis* Osbeck). The researchers found that, compared to wild-type oranges, the fungus-resistant mutant showed a decrease in both fatty acid synthesis and surface wax production, as well as an increase in synthesis of jasmonic acid, a plant hormone involved in anti-microbial defenses. The mutant seems to redirect production of fatty acids to make more jasmonic acid—and it’s this hormone that mediates the plant’s strong tolerance to fungal pathogens. This information could help agronomists breed other varieties of pest-resistant oranges that require less chemical fungicide under field and storage conditions.

## Introduction

Natural and artificial selections that exploit the rich crop genetic diversity can generate new stress-resistant varieties. The exploitation of naturally resistant varieties with elite agronomic traits can facilitate the development of sustainable agriculture by reducing the use of chemicals and enhancing the understanding of crop resistance to environmental stress^1,2^. For instance, the environmental adaptability of rice, tomato, and potato was enhanced by the natural variation in plant secondary metabolism, surface cuticle formation, and hormone signaling^2–5^. Therefore, studying and utilization of natural variations effectively facilitate the understanding in the crop defense.

As sessile organisms, plants constantly encounter a range of adverse conditions including pathogens, which greatly affect the growth, development, and productivity of crops. During the past two decades, our understanding of plant defense strategies including the constitutive and induced responses to pathogens was significantly enhanced by studies of natural variations^6,7^. Plant defense responses to pathogens involve complex gene networks that are closely associated with the morphological structure, physiological metabolism, and hormone signals^3,7,8^. The first constitutive barriers against pathogens are plant surface structures, such as the cuticle^8,9^. The cuticle is mainly composed of (i) cutin, which is formed by the polymerization of C16 and C18 hydroxy and epoxy-hydroxy fatty acids (FAs) and glycerol^9–11^, and (ii) cuticular wax, which is formed by very-long-chain fatty acids (VLCFAs; C20 to C34) and their derivatives together with secondary metabolites, such as triterpenoids^12,13^. The cuticle also has functions in sensing of signals associated with systemic acquired resistance (SAR)^14,15^. The components of cuticle can inhibit or activate the defense responses^8,9^. Indeed, some variations lead to robust compensatory responses against pathogens by (i) helping to induce a rapid oxidative burst that activates the hypersensitive cell death response (HR)^8,16^, (ii) inducing increases in the levels of antimicrobial peptides or PR gene expression^17^, and (iii) reducing the signals perceived by invading pathogens^18^. Once pathogens penetrate or circumvent the passive defense barrier, a series of highly coordinated changes are induced at the cellular level partially depending on hormonal signals. Jasmonic acid (JA) is a key component of the signalling system that responds to necrotrophic pathogens by inducing the expression of the plant defensin *PDF1*.*2*^19,20^. The jasmonate precursor cis (+)-12-oxo phytodienoic acid (OPDA) and JA are successively derived from the oxidation of linolenic acid (C18:3 FA) in plastids and peroxisomes. Conjugation reactions occur in the cytoplasm, generating active jasmonate, namely jasmonoyl-isoleucine (JA-Ile), and a transported form of jasmonate, namely methyl jasmonate (MeJA)^20^. The natural variation in *allene oxide synthase2* (*AOS2*) in potato affects the level of jasmonate and influences pathogen resistance in *Arabidopsis*^3^. The inhibitors of JA synthesis pathway are diethyldithiocarbamic acid (DIECA, an AOS inhibitor), ibuprofen (IBU, a lipoxygenase inhibitor) and n-propyl gallate (PG, a less specific inhibitor of both *LOX* and allene oxide cyclase (AOC))^20–22^. The orthologs of *Arabidopsis 13-LOXs*, *AOC*, and *AOS* are actively present in citrus^23,24^. Exogenous application of JA biosynthesis inhibitors and MeJA impact the plant responses to fungal stresses^22,24,25^. Although JA and the cuticle are both derived from FA synthesis in plastids^26^, few natural variations are known to affect both the cuticle and jasmonates. Thus, few studies have applied such variations to study plant defense. New biological resources and constant improvements of molecular tools are needed to dissect the defense mechanisms because the specific defense responses of plants are such complicated processes. Plant metabolic networks can help to organize omics datasets and to unravel the molecular regulatory network of fruit ripening or senescence in crops, such as tomato^27^, *Carica papaya*^28^ and citrus^29^. Accordingly, application of plant metabolic networks to study specific natural variations should be an effective strategy to investigate the defense mechanisms of plants.

As one of the seedless citrus crops, ‘Newhall’ navel orange (*Citrus sinensis* Osbeck) is usually subjected to commercial treatments to improve its resistance to pathogens and increase fruit glossiness before marketing. Approximately 15 years ago, a naturally occurring mutant (MT) of ‘Newhall’ navel orange was found to have strong pathogen resistance and a glossy surface after three generations of grafting. Thus, this MT can simplify the commercial postharvest processes of citrus fruit, showing important socioeconomic potential. The similar phenophase when grown in the same orchard, and identical genetic background of MT and the wild-type cultivar (WT) was verified by field observation and simple sequence repeat (SSR) and expressed sequence tags (EST)-SSR analysis, respectively (Supplementary File 1). MT was further identified by experts of horticulture and plant protection and registered as a new variety of crops of the People’s Republic of China (“Gannan NO.1”, No.CNA20130122.4). The strong resistance of MT to pathogens was confined to field observation, while there has been no systematic research on its defense mechanism. In this study, MT was characterized by comparing its biochemical and physiological properties with those of WT, especially its resistance to postharvest fungi. We aimed to reveal the biological basis of the strong fungal tolerance of MT for its potential economic applications.

## Results

### Assessment of the defense against pathogens in MT

**Fig. 1:**
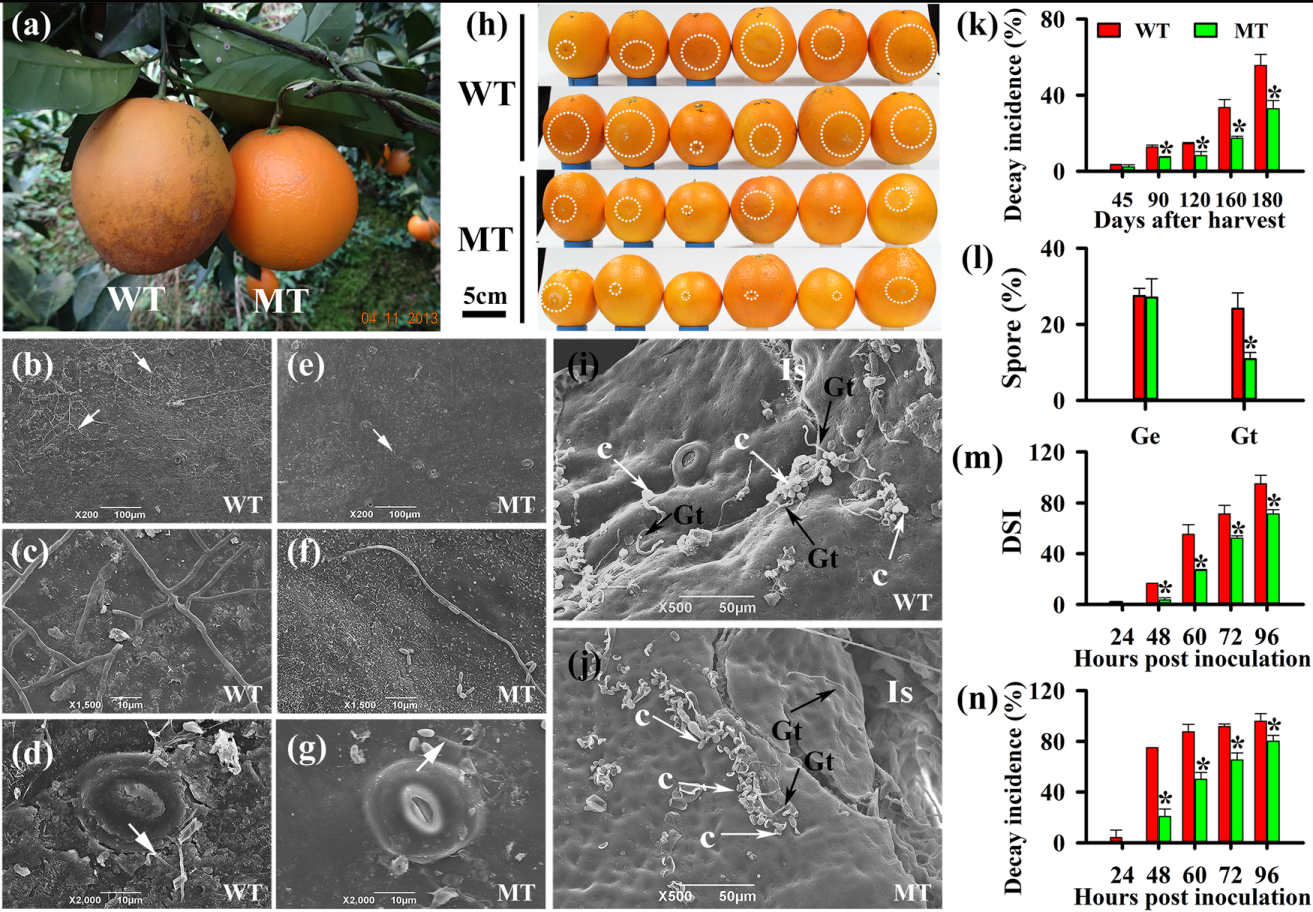
A comprehensive description of the protection against pathogens of MT and WT fruit. **a** Phenotypic comparison of the MT and WT fruit from adjacent trees in the same orchard. SEM analysis of WT (**b**–**d**) and MT (**e**–**g**) fruit surfaces. The white arrows indicate hyphae on WT and MT fruit. **h** Phenotype, **m** disease severity index and **n** decay incidence of WT and MT fruit infected with *P*. *digitatum*. The white arrows indicate hyphae on WT and MT fruit. The white circle indicates the lesion size of infected fruit. Pre-infection structure of *P*. *digitatum* on WT (**i**) and MT (**j**) fruit surfaces detected by SEM. **k** Fruit decay incidence during the ambient storage. **l** The rate of spore germination (Ge), and the percentage of germinated spore with germ tubes ≥ 20 μm in length (Gt) of *P*. *digitatum* within 12 h in WT and MT fruit. *Significant difference (*P* < 0.05, Duncan’s test). Data are presented as means ± SE (*n* = 3). DSI disease severity index, c conidia, Is inoculation site

We found a MT with an enhanced tolerance to fungal pathogen infections in the orchard, postharvest-storage, and artificial inoculation conditions. Under field conditions, MT was more tolerant to several types of disease caused by *Capnodium citri* and *Colletotrichum gloeosporoides*, as indicated by the visible increase in blemishes on the WT fruit relative to the MT fruit on an adjacent tree (Fig. 1a). Scanning electron microscopy (SEM) analysis of microbial pathogen status demonstrated that fewer hyphae and small plaques were present on the surface of MT fruit than that of WT fruit (Fig. 1b–g). Based on these results, we decided to test the decay incidence of the MT and WT fruit, which were subjected to individual commercial packaging for avoiding cross-infection of pathogens, and stored in a ventilated warehouse (temperature: 12– 20 °C; relative humidity (RH): 85–90%). From 45 days after harvest (DAH) to 180 DAH, the decay incidence of WT fruit rapidly increased from 3.37% to 55.65% in contrast to that of MT fruit which only increased from 2.26% to 32.87% (Fig. 1k). In citrus, the green mold caused by *Penicillium digitatum* (*P. digitatum*), blue mold caused by *Penicillium italicum* (*P. italicum*), and sour rot disease caused by *Geotrichum candidum* (*G. candidum*) account for the vast majority loss in fruit after harvest. To further compare the protection against pathogens of MT and WT fruit, artificial inoculation tests were conducted with *P. digitatum*, the most devastating and typical postharvest pathogen of citrus. We found that from 48 h post inoculation (HPI) to 96 HPI, the disease severity index (DSI) of MT increased from 4.78 to 71.43 in contrast to that of the WT which increased from 16.67 to 95.24 (Fig. 1m). Similarly, the decay incidence of MT was significantly lower than that of WT at 48 HPI (28.83% vs. 75.00%), 72 HPI (65.38% vs. 91.37%), and 96 HPI (80.12% vs. 100.00%) (Fig. 1n). We further examined pre-infection structure formation including the spore germination and differentiated germ tube of *P. digitatum* on MT fruit surface at 12 HPI, when spore germinated and began to infect the fruit. Approximately the same rate of spore germination (Ge, 24.11%) but a lower rate of spore with differentiated germ tubes (Gt, 10.87%) were observed in MT relative to in WT at 12 HPI (Fig. 1i, j, l), which were consistent with the results of pre-infection structure formation in MT at 24 HPI (Supplementary Figure S1a). Additionally, inoculation experiments with two additional major fungal pathogens of citrus *G. candidum* and *P. italicum* generated similar results (Supplementary Figure S1b, c). Taken together, these results confirmed the strong tolerance of MT to fungal pathogens, particularly the postharvest pathogens of citrus.

### Transcriptome and metabolome analysis of MT fruit

**Fig. 2:**
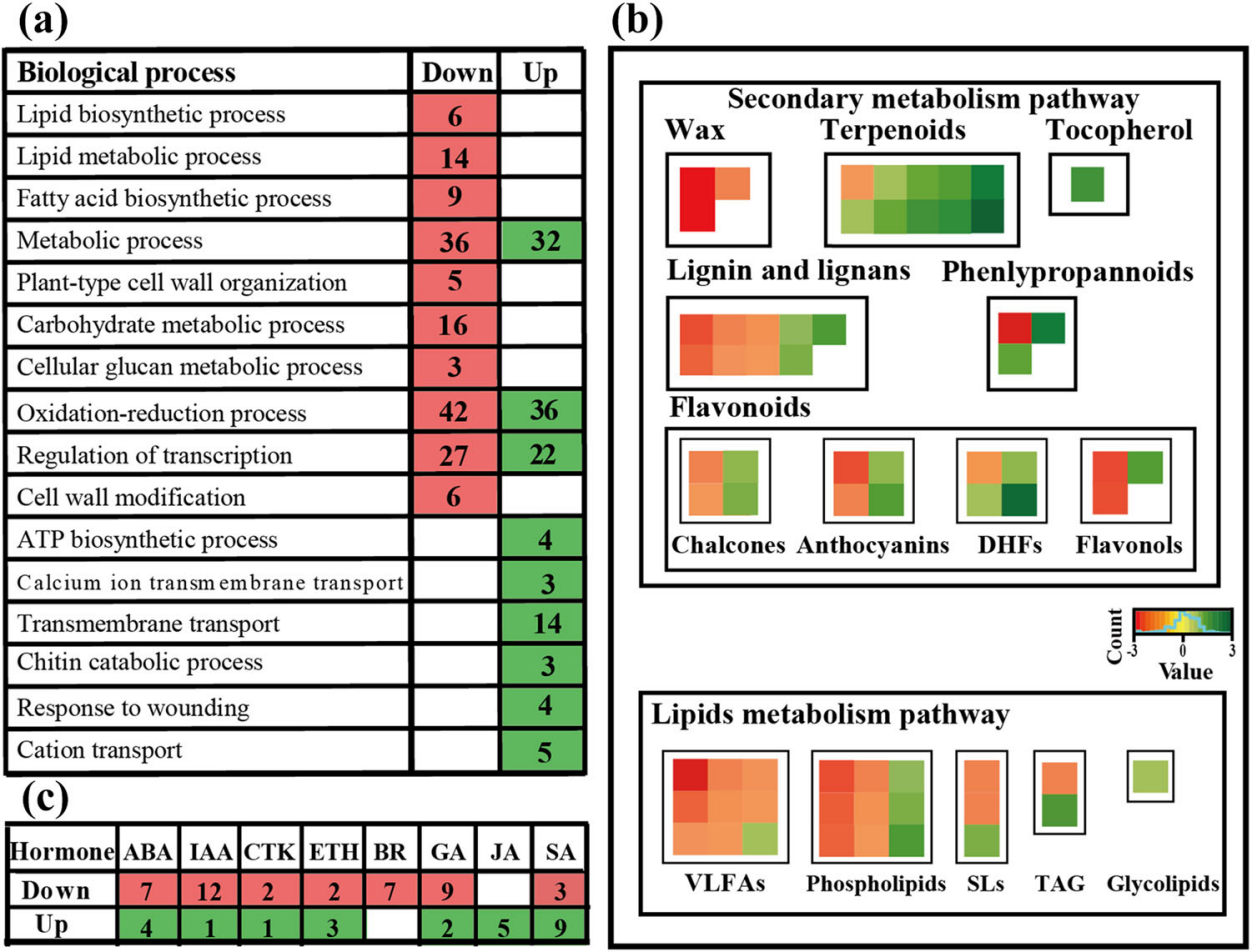
Overview of transcriptome analysis. **a** Functional categorization of the genes with significant transcriptional changes in WT and MT for biological process based on Gene Ontology annotations. **b** Schematics of the lipid and secondary metabolite pathways using the MapMan visualization platform. **c** Expression of genes involved in hormone signaling and response. The numbers in the red and green squares indicate the number of genes decreased or increased expression in MT fruit, respectively. DHFs dihydroflavonols, SLs sphingolipids, TAG triacylglycerol

To clarify the underlying mechanisms of the strong fungal pathogen tolerance in MT fruit, pericarps of WT and MT were collected and subjected to transcriptomic and metabolomic analyses. Using the *Citrus sinensis* genome as a reference, RNA-seq revealed that a total of 20,596 annotated genes were expressed in both WT and MT fruit. Compared with WT, the expression of 560 and 416 genes decreased and increased in MT, respectively (Supplementary File 2). A total of 20 differentially expressed genes (DEGs) were randomly selected from the RNA-seq analysis and their expression levels were independently quantified using qRT-PCR. The results indicated that the RNA-Seq data were reliable (Supplementary File 3). The GO term enrichment tests with these RNA-Seq data revealed that lipid metabolic and biosynthetic processes as well as FA biosynthesis process were significantly repressed in MT (Supplementary File 4, Fig. 2a). Similar results were obtained from a MapMan analysis (Fig. 2b). Furthermore, we identified 68 hormone-related DEGs, which were assigned to eight major categories of phytohormones (Supplementary File 5). Five genes related to JA biosynthesis, response, and signal transduction were substantially activated in MT, such as a putative chloroplast lipoxygenase (11-fold change) (Supplementary File 5, Fig. 2c).

A total of 193 metabolites were putatively identified in the WT and MT pericarps using HPLC-QTOF-MS and GC-MS, including lipids, polyphenols, flavonoids, terpenoids, hormones, and other metabolites (Supplementary File 6). A principal component analysis (PCA) of these metabolite data indicated a significant difference in the metabolome of MT relative to that of WT (Supplementary Figure S2). In MT, we observed a significant accumulation of flavonoids (such as 3,3′,4′,5,6,7,8-heptamethoxyflavone and narirutin), and at least two-fold increase in the levels of polyphenols (such as sinapic acid and 5-hydroxyconiferaldehyde) (Supplementary Figure S3). More importantly, JA was significantly accumulated in MT, but the contents of ABA, SA and auxin, and the ethylene production were unchanged (Supplementary File 6). This metabolic profile is indicative of an active defense response in MT.

### Network analysis of the citrus metabolic pathway

**Fig. 3:**
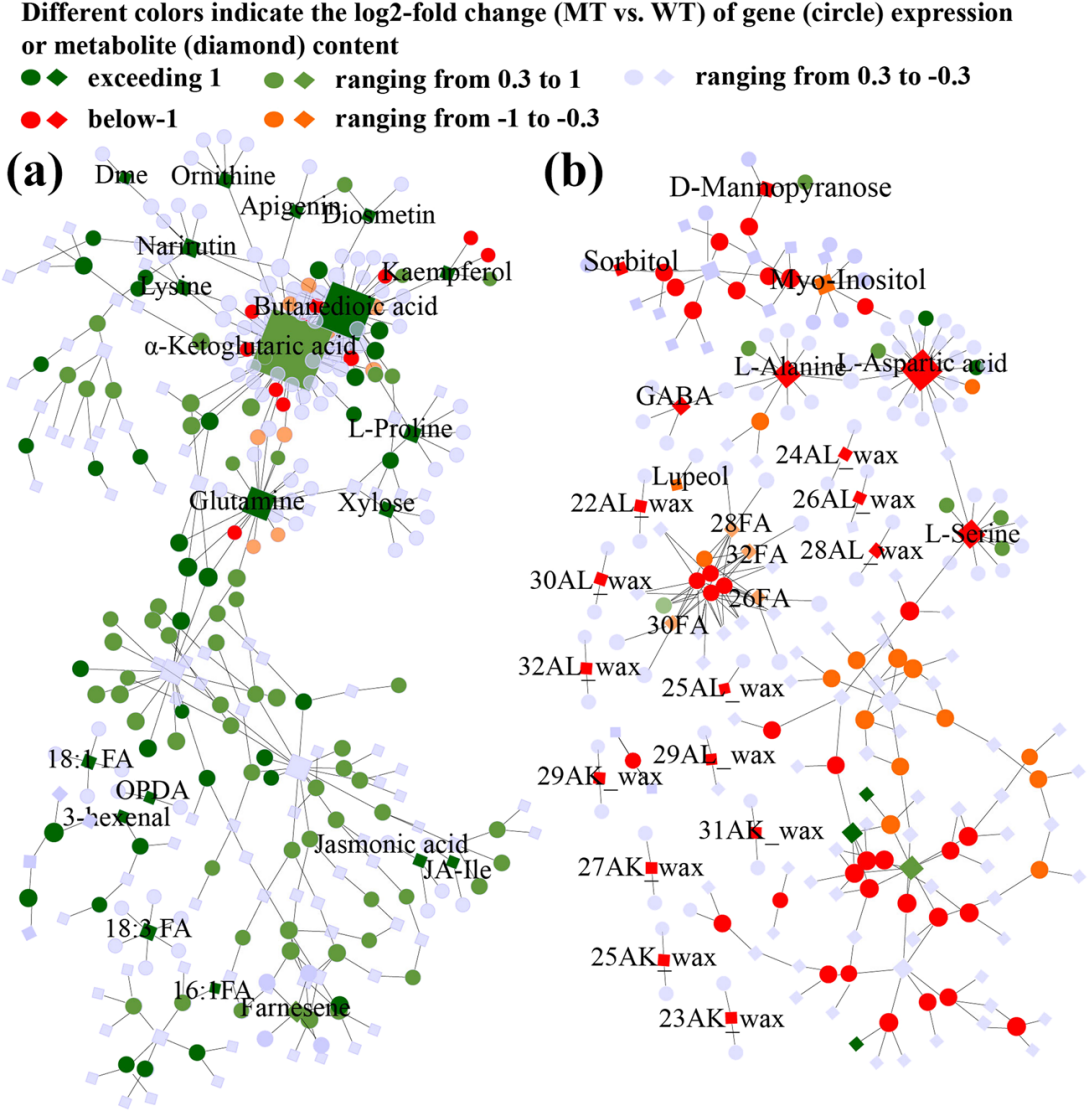
Correlations between transcriptome and metabolite data in CitrusCyc2.0. The network including increased- (**a**) and reduced- (**b**) sub-network was constructed using the absolute value of log2 fold change of DEGs (MT vs. WT, 0.3 or greater) and their first-level linkages from CitrusCyc2.0. The size of the diamond or the circle represents the central hub of metabolites or genes in the network. The numbers before FA (fatty acid), AL (aldehyde) and AK (alkane) indicate the length of carbon chain. Dme 5-demethylnobiletin

To integrate the transcriptomic and metabolomic datasets, an updated genome-scale metabolic network (CitrusCyc2.0) was constructed, which contains information from 2530 metabolism-related genes and 2370 metabolites (Supplementary File 7). We mapped the 20,596 genes and the 193 metabolites identified in this study into CitrusCyc2.0 (Supplementary Figure S4). We focused on the metabolism pathways including the nodes with high degrees of connection and BC of CitrusCyc2.0. The top five pathways with the highest correlation between the transcriptional and the metabolic data were analyzed in CitrusCyc2.0 (Table 1). A fine network was extracted according to the DEGs (MT vs. WT log_2_ fold change ≥ 0.3) and their first-level of linkage and was divided into the elevated sub-network (sub-network a) and the decreased sub-network (sub-network b). We analyzed the highly coordinated pathways in the transcriptomic and metabolic data in the two sub-networks (Fig. 3, Supplementary File 8 and Table 1). The data indicated that the VLCFAs elongation and wax synthesis pathways in MT fruit were coordinately linked to sub-network b (Fig. 3b). Pathways related to unsaturated fatty acids were also enriched in sub-network a (Fig. 3a). More importantly, the levels of jasmonates and α-linolenic acid were highly coordinated with the expression of jasmonate-associated genes in sub-network a (Fig. 3a). JA-induced metabolites such as 3′,4′,3,5,6,7,8-heptamethoxyflavone and 5-demethylnobietin and proline were enriched in sub-network a (Fig. 3a). A total of 10 genes involved in VLCFAs elongation and wax synthesis and JA synthesis are selected from sub-network for qRT-PCR test (Supplementary File 4). The expression levels of genes such as *KCS1*, *KCS6*, and *KCS10* (β-ketoacyl-CoA synthases), *CER1* (VLC-aldehyde decarbonylase) and *CER4* (fatty acyl-CoA reductase) were decreased in MT fruit, which were consistent with the SEM observation of considerably fewer wax crystals on MT fruit surface (Supplementary File 9, Fig. 1b–g). The increase in gene expression levels of JA synthesis such as *LOX3* and *AOS* was consistent with JA level in MT. These results indicated the high reliability of this network. Based on the results from the network analysis, we carried out the removal of wax and peeling of flavedo, and exogenous application of JA elicitors and inhibitors to WT and MT fruit to further clarify the functions of cuticular wax and JA in the tolerance of MT.

**Table 1:** Top 5 pathways with the highest correlation between the transcriptional and the metabolic data in CitrusCyc2.0

Pathway names	KEGG ID
α-Linolenic acid metabolism	map00592
Isoflavonoid biosynthesis	map01040
Cutin, suberine, and wax biosynthesis	map00073
Pantothenate and CoA biosynthesis	map00770
Biosynthesis of unsaturated fatty acids	map01040

### Relation between wax layer and the defense of MT fruit

**Fig. 4:**
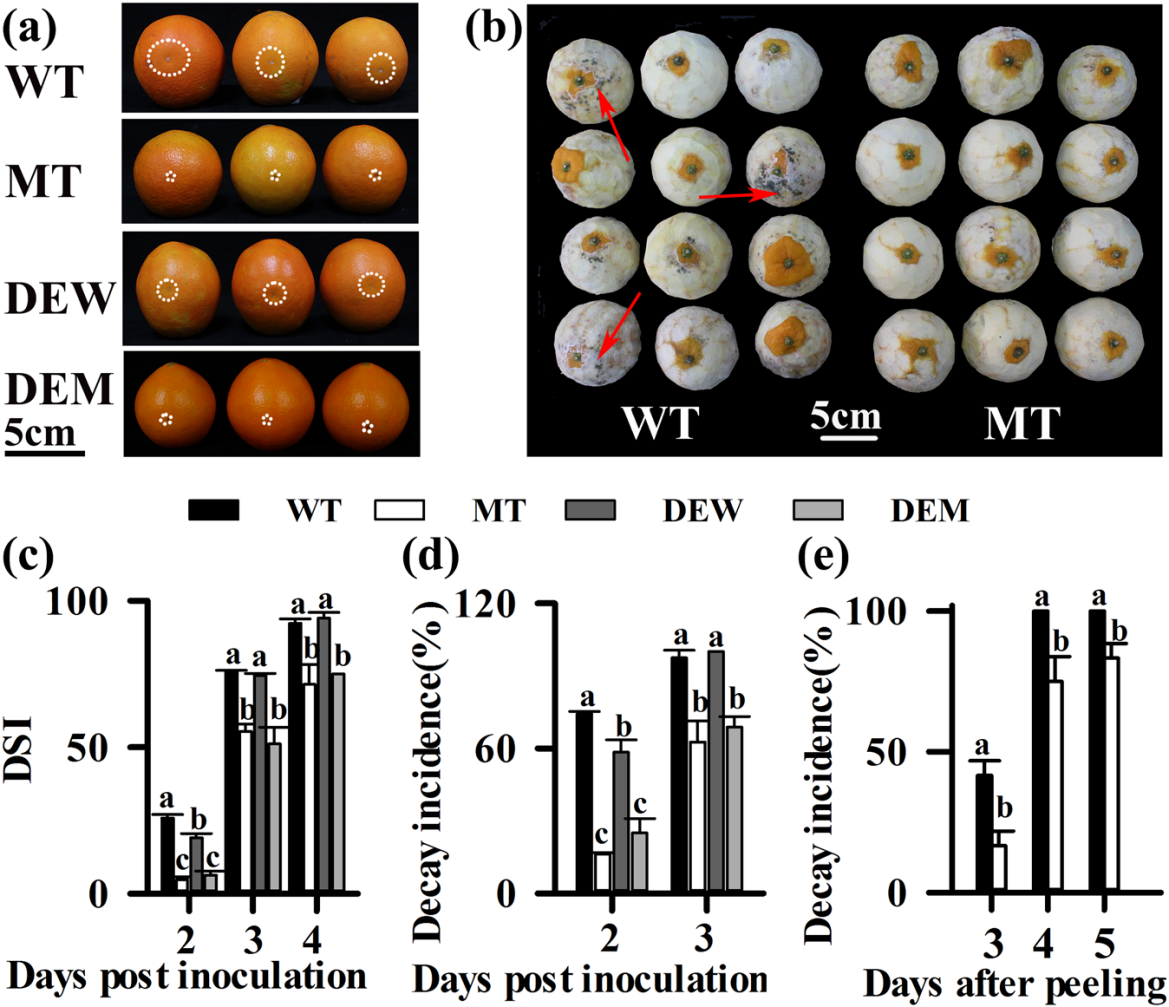
Inoculation experiments of WT and MT fruit with different treatments. **a** Phenotype, **c** disease severity index and **d** decay incidence of the WT, MT, and de-wax treated WT (DEW) and MT (DEM) fruit after the *P. digitatum* infection. The white circle indicates the lesion size of infected fruit. **b** Phenotype and **e** decay incidence of WT and MT fruit after peeling of flavedo. The red arrows indicate the infected zones of pericarp. Different letters indicate statistically significant differences (*P* < 0.05, Duncan’s test). Data are presented as means ± SE (*n* = 3)

We found that the deficiency of wax layer in MT fruit was not the direct reason for the enhanced tolerance to *P. digitatum*. The wax load of MT fruit was reduced by 81.34% compared with that of WT fruit. The majority of aliphatic wax compounds including aldehydes, VLCFAs, alkane and primary alcohol were decreased in MT, but the loads of non-aliphatic triterpenoids were not discernibly altered in MT (Supplementary Figure S5a, b). Based on the analysis of transmission electron microscopy (TEM), the thickness of the cutin layer, the content and composition of cutin monomers were similar in MT and WT fruit (Table 2, Supplementary Figure S5f and S6). To test the role of cuticular wax in the pathogen tolerance of MT fruit, we treated WT and MT fruit with Arabic gum to remove wax while maintaining the cutin layer. The WT, MT, de-waxed WT (DEW), and de-waxed MT (DEM) fruit were inoculated with *P. digitatum* (Fig. 4a). The DSI of DEM increased from 6.25 at 2 DPI to at 75.00 at 4 DPI, which was similar to that of MT (Fig. 4c). The DEW resembled the MT in terms of small amounts of epicuticular wax. The DSI of DEW was significantly higher compared with MT fruit at 2 DPI (19.05 vs. 4.76), 3 DPI (74.40 vs. 55.36) and 4 DPI (94.05 vs. 71.43) (Fig. 4c). Although the DSI of DEW was significantly lower than that of WT (25.72 at 2 DPI), the DSI of DEW was not significantly different from that of WT at 3 DPI (74.40 vs. 75.58), and at 4 DPI (94.05 vs. 92.26) (Fig. 4c). Consistently, the decay incidence of MT (62.5%) was significantly lower than that of WT (97.44%) and DEW fruit (100%) at 3 DPI (Fig. 4d). Therefore, DEW fruit were not as tolerant as MT fruit. Furthermore, at 4 days after peeling of flavedo, the decay incidence of MT (75%) was significantly lower than that of WT (100%) (Fig. 4b, e). A storage experiment showed similar results in MT and WT with wax removal by chloroform (Supplementary File 10). All the results suggested that some other factors rather than the loss of wax contribute to the strong tolerance of MT to *P. digitatum*.

**Table 2:** Characterization of the fruit surface in WT and MT fruit. Three biological replicates were examined for each variety

Variables (abbreviations, units)	WT (Mean values ± SE bars)	MT (Mean values ± SE bars)
Cutin thickness (μm)	3.51 ± 0.08	3.55 ± 0.12
Cutin load (μg cm^−2^)	204.93 ± 28.74	219.16 ± 23.35
Cell wall thickness (μm)	1.04 ± 0.09	1.00 ± 0.03
Epidermal cell area (μm^2^)	76.30 ± 2.50	81.60 ± 2.57
Contact angles	111.86 ± 3.67	82.35 ± 1.92*

Mean values and SE are indicated

*Significant difference (*P* < 0.05, Duncan’s test)

### Influence of *P. digitatum* on JA biosynthesis and signaling pathway in MT

**Fig. 5:**
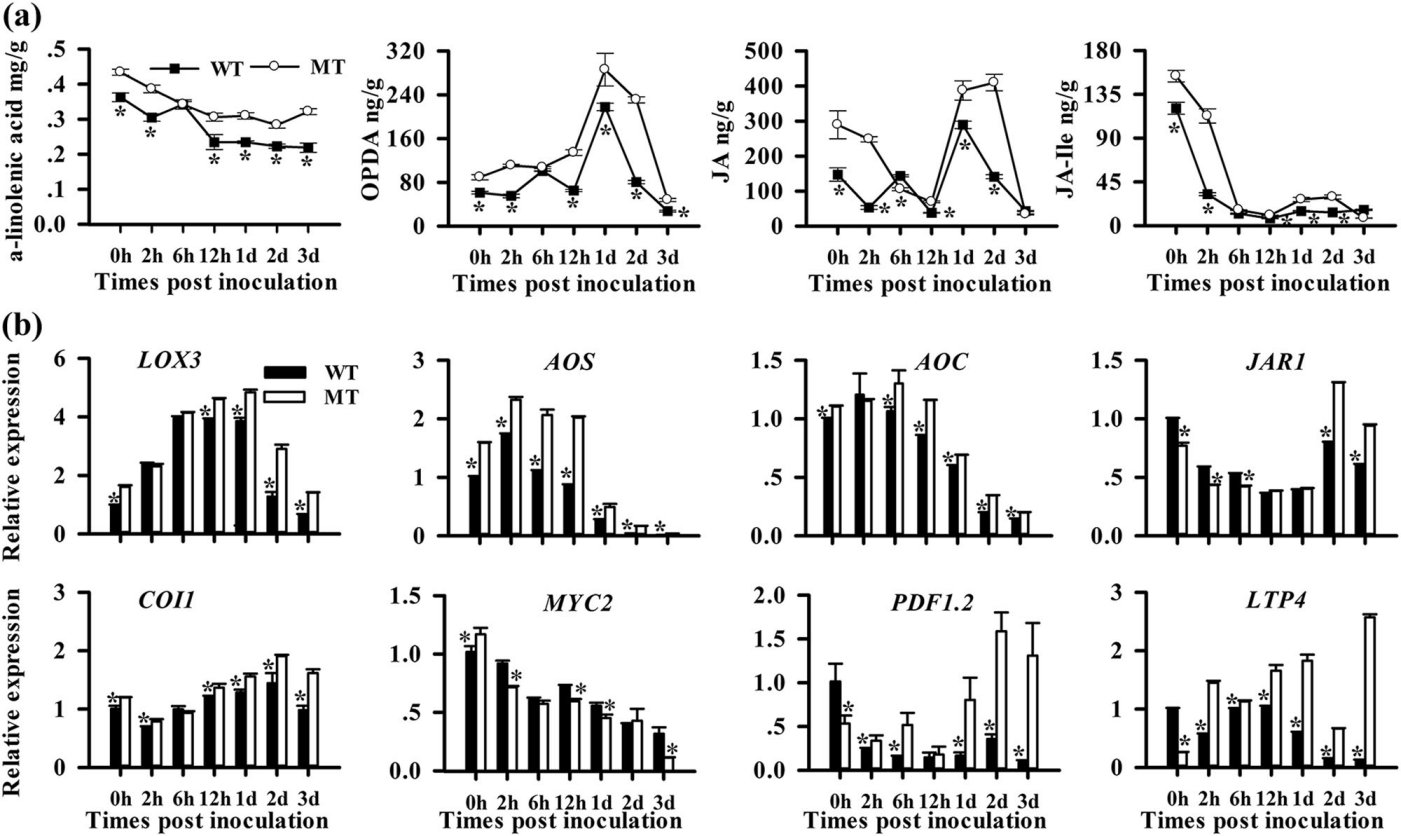
Characterization of JA-mediated defense responses in WT and MT fruit after inoculation. **a** Levels of α-linolenic acid and jasmonates. **b** Expression levels of JA synthesis, signaling and response genes. Different letters indicate statistically significant differences (*P* < 0.05, Duncan’s test). Data are presented as means ± SE (*n* = 3)

To assess whether JA biosynthesis was induced by *P*. *digitatum* infection, we quantified the levels of α-linolenic acid and jasmonates (including OPDA and JA-Ile) in MT and WT fruit. In MT, we observed the higher *P. digitatum*-induced gene expression and metabolite levels associated with JA biosynthesis and signaling. During the fungal infection, similar accumulation trends but clear quantitative differences in the accumulation of α-linolenic acid and jasmonates were observed in both MT and WT (Fig. 5a). The content of α-linolenic acid in MT gradually decreased from 0.43 mg g^−^^1^ (0 HPI) to 0.32 mg g^−^^1^ (3 DPI), but dramatically decreased from 0.36 mg g^−1^ (0 HPI) to 0.21 mg g^−^^1^ (3 DPI) in WT. The OPDA content of MT started to increase at 2 HPI (110.92 ng g^−^^1^) to 2 DPI (230.97 ng g^−^^1^), which was higher than that in WT (80.70 ng g^−^^1^) (Fig. 5a). Consistently, the content of JA-Ile decreased after the initial infection in MT but later increased from 10.67 ng g^−^^1^ at 12 HPI to 26.90 ng g^−^^1^ at 2 DPI. In contrast, we observed almost no change in the level of JA-Ile in WT during the same period. Similarly, the JA content of MT decreased after the initial infection but rose from 69.63 ng g^−^^1^ at 12 HPI to 387.33 ng g^−^^1^ at 1 DPI, and then peaked to 410 ng g^−^^1^ at 2 DPI. At 2 DPI, the JA content was approximately 3-fold higher in MT than in WT (143.33 ng g^−^^1^) (Fig. 5a). We monitored the expression of the genes associated with the JA biosynthetic and signaling pathways in MT and WT after the fungal inoculation. The expression levels of the JA biosynthesis genes *AOS*, *AOC*, and *LOX13*, and JA signaling genes *COI1* (coronatine insensitive1) and *JAR1* (JA-resistant 1) were much higher in MT, particularly at 12 HPI (Fig. 5b). Although the expression levels of *MYC2* significantly decreased in MT, those of *PDF1.2* and *LTP4* (lipid transfer protein 4) and the PTI marker *GST1* were much higher in MT at 1 DPI (4.80fold-, 3.04-fold, and 1.26-fold changes, respectively; Fig. 5b). In MT, during the infection, we observed a significant rise in the levels of stress-associated metabolites, such as *α*-tocopherol and polymethoxylated flavonoids (PMFs), especially 5-dimethylnobietin (Supplementary Figure S8). Above-mentioned results proved that the JA pathway was more significantly induced by *P. digitatum* in MT.

### Exogenous application of JA elicitors and inhibitors to citrus fruit

**Fig. 6:**
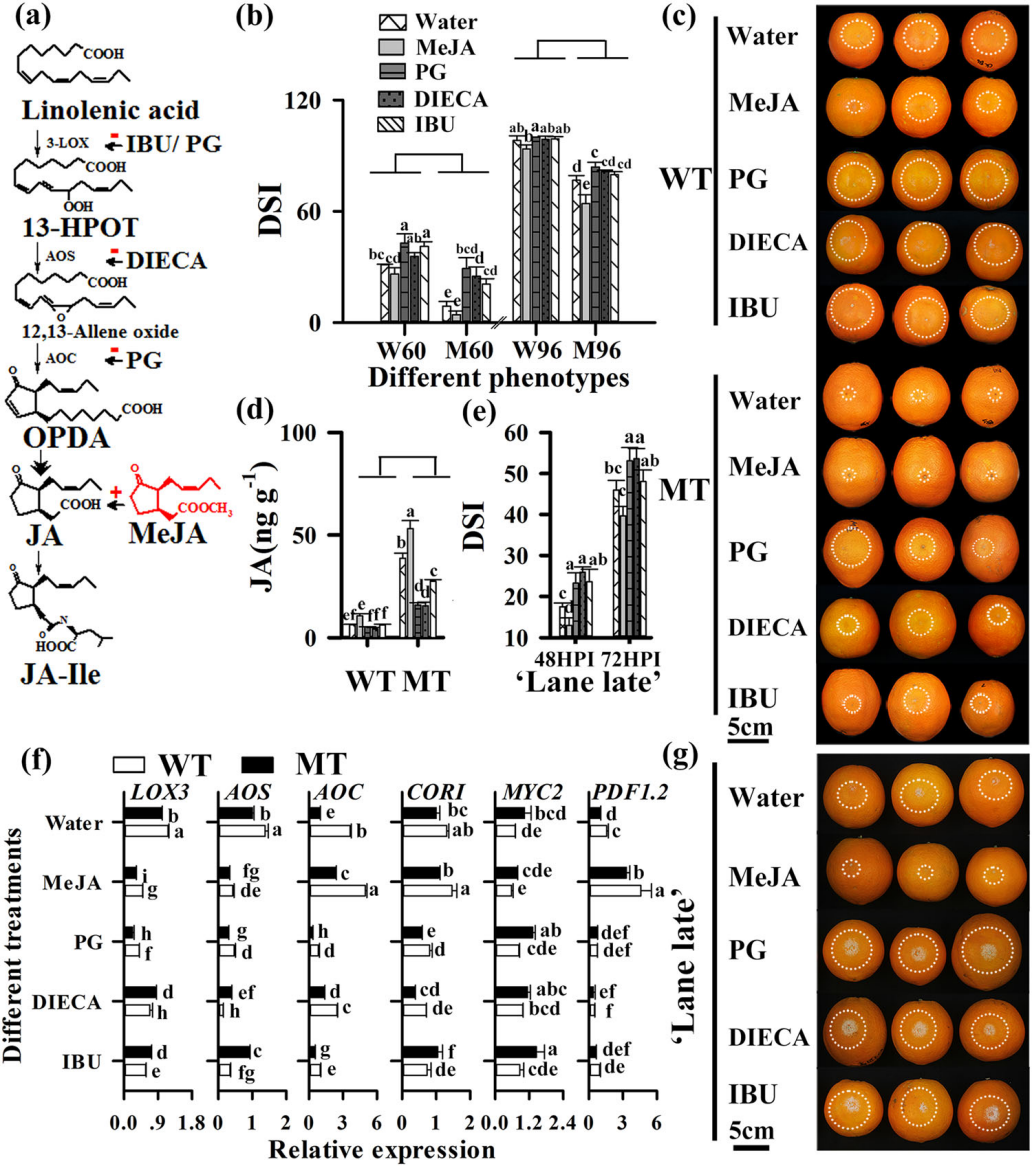
Jasmonic acid plays a positive role in protection against *P. digitatum* in MT fruit. **a** The Vick-Zimmerman pathway of JA biosynthesis from a-linolenic acid. Representation of the pathway with an indication of which steps of the signal-transduction pathway is affected by different elicitors (+) and inhibitors (−). Ibuprofen interferes with lipoxygenase activity, diethyldithiocarbamic acid inhibits AOS activity, and n-propyl gallate inhibits both lipoxygenase and AOC activity. **b** DSI and **c** Phenotype of treated WT and MT fruit at 60 and 96 HPI. **d** Level of JA, and **f** expression levels of JA synthesis, signaling and response genes in treated WT and MT fruit at 12 h post *P. digitatum* infection. **e** Phenotype and **g** DSI of ‘Lane late’ navel orange after *P. digitatum* infection under different treatments. The white circle indicates the lesion size of infected fruit. Different letters indicate significant differences among WT, MT and their corresponding treatments at 60 and 96 HPI, respectively (*P* < 0.05, Duncan’s test). Data are presented as means ± SE (*n* = 3). The 60 and 96 before W (WT) and M (MT) indicate hours post *P. digitatum* infection (HPI)

To test whether the observed tolerance is dependent on JA in MT, the WT and MT fruit were treated with MeJA and JA inhibitor (PG, DIECA, and IBU) (Fig. 6a). The DSI of water treated WT (31.19) was higher relative to that of MeJA treated WT (22.14), but significantly lower than that of PG (42.86) and IBU (41.07) treated WT at 60 HPI (Fig. 6b, c). At 96 HPI, the DSI values of water treated WT (98.21) were not significantly different from those of MeJA and JA inhibitors treated WT (Fig. 6b). However, the DSI of water treated MT (76.78) was significantly higher than that of MeJA treated MT (64.29), but significantly lower than that of PG treated MT (83.93) at 96 HPI (Fig. 6b). Consistent with the DSI values, the decay incidence of MeJA-treated MT (27.50%) was significantly lower than that of PG, DIECA and IBU treated fruit of MT and WT at 60 HPI (Supplementary Figure S7). Furthermore, at 12HPI, the expression levels of *LOX3* and *AOS* were decreased by 56.68% and 78.01% in MeJA-treated MT (relative to water treated MT), and 77.51% and 57.76% MeJA-treated WT (relative to water treated WT) in a feedback way, which was similar to that of *MYC2*. However, the values of *AOC*, *COR1*, and *PDF1.2* were increased to 4.96, 1.37, and 4.58-fold changes in MeJA treated MT relative to water treated MT, which was similar to the case of MeJA treatment in WT (Fig. 6f). As for JA inhibitor treatment, the expression levels of *LOX13*, *AOS,* and *AOC* decreased in MT after PG (34.37, 66.09, and 77.53%), DIECA (58.53, 89.67, and 72.81%) and IBU (49.11, 75.46, and 31.94%) treatment relative to water treated MT, and similar results were obtained in WT (Fig. 6d). Consistently, the content of JA in MeJA treated WT (10.68 ng g^−1^) was higher than that in water treated WT (6.40 ng g^−1^) (Fig. 6d). The JA content of MeJA treated MT (52.98 ng g^−1^) was higher than that in water (38.61 ng g^−1^), PG (15.98 ng g^−1^), DIECA (15.61 ng g^−1^), and IBU (27.38 ng g^−1^) treated MT (Fig. 6d). Therefore, exogenous application MeJA or JA inhibitors on MT and WT fruit stimulates or limits endogenous JA level and expression of JA responsive genes.

To examine the effect of JA on the induced defense in citrus fruit, ‘Lane late’ and *Citrus clementina* fruit were treated with MeJA and JA inhibitors (Fig. 6a). The water treated (17.53 at 48 HPI, 45.95 at 72 HPI) ‘Lane late’ showed a higher DSI relative to the MeJA (12.98 at 48 HPI, 39.68 at 72 HPI) treated fruit. However, the DSI values of PG, DIECA, and IBU treated fruit reached 23.21, 25.89 and 23.57 at 48 HPI, and 53.08, 53.57 and 48.02 at 72 HPI, respectively (Fig. 6e, g). The decay incidence of MeJA (63.64%) treated fruit were significantly lower than those of water (86.37%) and JA inhibitor-treated fruit at 48 HPI (Supplementary Figure S8). We also found MeJA treated fruit of *Citrus clementina* generated similar results (Supplementary Figure S8). All the results of the exogenous application of JA elicitor and inhibitors to citrus fruit indicated that the JA-mediated defense of MT fruit significantly contributes to the strong tolerance to *P. digitatum*.

## Discussion

This study highlights the elite agronomic traits of strong tolerance to fungal pathogens in glossy MT. Our results indicate that the enhanced JA-mediated defense contributes to the tolerance of MT to fungal infections. Recently, another glossy orange fruit ‘Ganqi 3’ was reported, and the wax of fruit was analyzed in ‘Ganqi 3’ and the relevant WT^30^. In this study, MT fruit exhibited significantly reduced aliphatic wax components and but the constant cutin components. Consistently, the decreased expression levels of genes such as *KCS1*, *KCS6*, *CER1*, *CER3*, *CER4*, and *MYB96* contributed to the wax deficiency in MT, which were similar to the results of Martin and Rose^9^ and Bernard and Joubès^12^. The alteration of wax, cutin, or both of them increase in the specular reflection or decrease in diffuse light reflection and give a glossy aspect to plant surface in *Arabidopsis* and tomato^11,12,31^. Therefore, brightness modification of MT is attributed to wax deficiency. One striking difference between ‘Ganqi 3’ and MT is that the tolerance to pathogens is not enhanced in ‘Ganqi 3’ fruit during the long-term storage^32^. These data provide evidence that a different mutation event may occur in MT and ‘Ganqi 3’.

### Possible biological mechanism against fungal pathogen infection in MT

To our knowledge, this is the first report of a wax-deficient MT of citrus exhibiting an enhanced defense against pathogenic fungi, a consistent observation that we repeatedly verified during five consecutive years (2012–2016). MT fruit exhibited a less hydrophobic and a more permeable surface, as indicated by their lower average contact angle (82.4°), faster weight loss, and more rapid carotenoid leaching (Table 2 and Supplementary Figure S6). A significant increase in the permeability of the cuticle, which is associated with the release of ROS and the accumulation of cuticle-derived product, is known to promote the defense against pathogens^8,15,16^. Regarding the release of ROS, when the hyphae began to invade the cells of the pericarps at 12 HPI, there were no significant differences in the accumulation of H_2_O_2_ in the fruit of MT and WT (Supplementary Figure S8). As for the cuticle-derived product, the cuticular structure of DEW fruit resembled that of MT fruit in terms of intact cutin layer and small amounts of epicuticular wax. The DEW and WT fruit were similarly tolerance to pathogens and in contrast, the DEW fruit was less tolerance to pathogens compared with MT fruit. We also found that spore germination was not inhibited by the wax deficiency in MT, which conflicts with reports indicating that epicuticular wax components are required for the germination of spores of fungal pathogens^18,33^. Furthermore, 4 days after peeling, the decay incidence of MT was significantly lower than that of WT, which was consistent with that of MT and WT with wax removal by chloroform during the storage. These results indicate that the loss of wax or generation of cuticular breakdown products is not required for the strong pathogen tolerance in MT, suggesting that the phenotype of MT is complex. Interestingly, we found the higher basal and *P. digitatum*-induced gene expression and metabolite levels associated with JA biosynthesis and signaling in MT. Exogenous application of MeJA and JA inhibitors respectively boosted and reduced the protection against *P. digitatum* in the ‘Lane late’, *Citrus clementina*, WT and MT fruit, which is consistent with the results of Rodriguez et al.^24^. Besides, the significant alteration of endogenous JA synthesis affected the tolerance of MT, but had no effect on that of WT at 96 HPI. JA and SA participate in the defense against necrotrophic pathogens and biotrophic/hemibiotrophic pathogens, respectively^6^. Negative interactions between JA and SA signaling pathways have been broadly documented^34^. During *P. digitatum* infection, the levels of SA and the expression levels of genes associated with SA synthesis and signaling decreased in MT compared with WT (Supplementary Figure S6). Together, in MT, an active JA-mediated defense contributes to the tolerance of *P. digitatum*.

### Possible mechanism between decreased wax and increased JA pathway in MT

The mechanism that activates the JA-dependent defense and improves the fungal tolerance in MT remains an open question. The JA-mediated defense might be rapidly provoked by the damage or wound, and enforce the response to opportunistic fungi infection^24^. Although a mechanical damage in fruit is required for *P. digitatum* invasion, the JA level in MT and WT inoculated with *P. digitatum* was higher than that in MT and WT inoculated with water, respectively (Supplementary Figure S10a). Additionally, the higher JA level of MT, with no visible injury at the mature stages, was consistent with the lower decay incidence. *Arabidopsis MYC2* (a helix-loop-helix-leucine zipper type TFs) positively regulates the wounding-responsive genes, but negatively regulates pathogen-responsive genes *PDF1.2*^19^. During the inoculation, the expression of *MYC2* gradually decreased, but that of *PDF1.2* significantly increased in MT. These results indicate that mechanical injury is not required for the significant accumulation of JA in MT compared with WT.

JA and cuticle are both derived from FA synthesis in plastids^26^. We found that the levels of VLCFAs with chains longer than C22 were reduced, but polyunsaturated fatty acids (PUFAs) such as C18:3 FAs and its derived JA were elevated in MT. The negative correlation between JA and wax synthesis was observed in MT. Most previous results showed that membrane lipids but not VLCFAs elongation and wax were investigated in JA-overproducer mutants, but that lipid and JA metabolism was altered in wax-deficient mutants^35–40^. Similar to the phenotype of MT, the *Osfae1*, *Atkcs1*, and *Atmyb30* mutants decreased in the VCLFAs but increased UFAs synthesis especially the C18:3 FA, which provides the procurer of JA accumulation and altered the resistance to fungal pathogens^12,35,41–43^. Therefore, we hypothesize that fatty acid metabolic flow between blocking VLCFAs elongation and then wax synthesis, and the activation of PUFAs and then jasmonates synthesis existed in MT. We also found that seven fatty acid desaturases (FADs) were activated, such as two putative chloroplast FADs (2.63 and 3.32-fold changes) in MT. To support this hypothesis, we treated the fruit of Hongkong kumquat (*Fortunella hindsii* Swingle) with metolachlor at 60 days after anthesis (Supplementary Figure S10b). After 2 weeks of 100 μM treatment, VLCFAs elongation of fruit was decreased, together with the increased C18:3 FAs synthesis, which is consistent with the results of rice, maize, and sorghum^44^. Similarly, it was reported that the cuticular lipids were altered and that the JA-mediated defense against fungal pathogens was improved simultaneously^45^. In addition, the normal permeability of the cuticle in the *rst1* MT indicates that the JA-mediated defense response and the permeability of the cuticle are independent processes^45^. MapMan visualization of the abiotic stress response showed that indeed, the signaling response to stress stimuli mediated by JA upstream of the MAPK pathway induced a more intense immune response in MT (Supplementary Figure S11). Consistent with these observations, the expression of defense response genes, such as *PDF1.2*, *GST1*, and LTPs, was elevated in MT. The expression of *PDF1.2* was changed in cuticular lipid MTs^19,35^. LTPs play a significant role in the formation of cuticle and the transport of jasmonates across membranes^8^. Additionally, liposoluble α-tocopherol and 5-dimethylnobiletin were significantly elevated in MT. These metabolites are specifically induced by JA signaling genes, and protect cells from free radicals and stabilize membrane structures^46^. Their high concentrations are usually correlated with a low decay incidence of *Citrus aurantium* by *P. digitatum*^46,47^. Apparently, in MT fruit, a physical barrier is removed by wax deficiency and a corresponding mechanism induces JA-mediated defense response, which contributes to an effective multifaceted defense against fungal pathogens.

In accordance with the development of modern and eco-friendly agriculture, MT decreases the use of chemical fungicides under the field and storage conditions and simplifies the commercial postharvest processes such as waxing and washing. To date, MT has been promoted as a new variety of major citrus growing areas of China. In this study, plant metabolic networks revealed that cuticular wax is significantly reduced and JA synthesis is significantly increased in MT. The glossy surface of MT is attributed to wax deficiency. We also found that the activation of JA synthesis contributed to the strong pathogenic fungi tolerance by the redirection of FA metabolism of MT fruit. Therefore, research on elite agronomic traits of strong tolerance to fungal pathogens and glossy surface of MT will bring about great socioeconomic benefits as well as facilitate the understanding of wax synthesis and defense response.

## Materials and methods

### Experimental materials

Commercially mature fruit of WT and MT was harvested from the same orchard using normal cultural practices in Anyuan County of Jiangxi province, China (GPS co-ordinate, 115.40W-25.13E) in six successive years from 2011 to 2016. Approximately 200 kg of fruit with a uniform size and free of visible injury were collected from each variety. Pericarps from eight fruit of each type were sampled with three biological replicates. These samples were immediately frozen in liquid nitrogen and stored at −80 °C for further metabolome and transcriptome analysis from 2011 to 2014. SEM, TEM, and light microscopy with Sudan IV staining were conducted in four successive years from 2011 to 2014. Fruit used for wax and cutin analysis was harvested from 2011 to 2013. The remaining fruit of both MT and WT was subjected to individual commercial packaging procedures and stored in a ventilated warehouse (recorded by the electronic precision long-time thermo-hygrograph (LGR-WSD20)) to test their postharvest resistance to pathogens from 2012 to 2016. For the exogenous MeJA and JA biosynthesis inhibitors treatment, ‘Lane late’ navel orange (*Citrus sinensis* Osbeck), and late-maturing *Citrus clementina* fruit was randomly harvested from a commercial orchard in Yichang (Hubei Provence, China), and the National Citrus Breeding Centre of China (Huazhong Agricultural University, Hubei Provence, China) in 2013, 2014, and 2017.

### DNA extraction and SSR analysis

The extraction of total DNA from WT and MT leaves and SSR analysis were performed as described by Cheng et al.^48^. Primer sequences are presented in Supplementary File 11.

### Microscopy analysis

About 3 mm^2^ of pericarps were excised from the fruit equatorial zone for SEM as described by Wang et al.^49^. The preparation of samples for TEM and the detailed inspections were conducted as described by Wang et al.^50^. The frozen sectioning technique was used for the production of 8 µm sections as described by Wang et al.^50^. Eight biological replicates were analyzed.

### Surface permeability and hydrophobicity measurements

According to Wang et al.^49^, eight fruit per phenotype were used to determine the rates of water loss. Based on the permeability of the cuticle to the level of chlorophyll^40^, we normalized the permeability of the cuticle to the level of carotenoid of mature orange fruit as described by Lee et al.^51^. This procedure was described in more detail in Supplementary File 11. To quantify the surface hydrophobicity^33^, approximately 4 cm^2^ of pericarp tissue was excised from the equatorial zone of six fruit and the contact angle was measured using the sessile drop techniques as described in Supplementary File 11. Three biological replicates were analyzed.

### Total RNA extraction and RNA-Seq analysis

Pericarp tissues, including the flavedo and the albedo tissues (three biological replicates each for WT and MT), were used for extracting total RNAs as described by Ma et al.^52^. The cDNA library was constructed and the products were qualified, quantified, and sequenced using an Agilent 2100 Bioanaylzer, an ABI StepOnePlus Real-Time PCR System, and an Illumina HiSeqTM2000, respectively.

### Detection and functional classification of DEGs

Raw data were filtered using a FASTX-toolkit. More than 80% of the bases had a quality score higher than 28. The filtered raw reads were aligned to the reference genome from *Citrus sinensis* using Tophat 2.08^52^. Cufflinks workflow and Interproscan were used for the detection and annotation of DEGs (*P* ≤ 0.05 and fold change ≥ 2), respectively. Hypergeometric probabilities were used to test the association of GO terms with DEG lists. GO terms with a corrected *P-*value < 0.05 were considered to be significantly enriched. To identify differentially expressed TFs^52^, we compared our datasets with 2256 citrus TFs in the PlantTFDB using the BLASTN software (Supplementary File 11).

To validate the reliability of the RNA-Seq data and the expression levels of stress response-related genes, quantitative real-time polymerase chain reaction (qRT-PCR) was conducted as described by Ma et al.^52^. Primer sequences are presented in Supplementary File 12.

### Extraction and analysis of cuticular lipids

Cuticular waxes were extracted with chloroform from ten fruit using the method of Wang et al.^50^. Three biological replicates were analyzed for each phenotype. Extracts were incubated in pyridine for 30 min at 50 °C, followed by treatment with bis-N, N-(trimethylsilyl) trifluoroacetamide (BSTFA) containing 1% trimethylchlorosilane (TMCS) (Sigma) for 40 min at 60 °C. After concentrating under N_2_, the samples were re-dissolved in chloroform and analyzed by GC-MS (Thermo Fisher, ISQII, USA) equipped with a flame ionization detector (FID) and an Agilent DB-1 column (30 m × 25 μm i.d. × 0.1 μm). Amounts of all detected compounds were assessed by comparisons with the internal standard.

The cutin membrane was isolated using three types of enzyme solutions (cellulase, pectinase, and hemicellulase, Sigma-Aldrich) from pericarp as described by Li-Beisson et al.^10^. Hundred milligram of cutin powder was depolymerized with 20 mL of 14% BF_3_/MeOH (Fluka) and then with 60 mL of saturated NaHCO_3_/H_2_O. The cutin monomers were extracted with 500 µL of chloroform using *n*-tetracosane (5 µg µL^−1^) as an internal standard. The subsequent procedures for the derivatization, re-dissolution, analysis, and identification were the same as the methods used for cuticular waxes described above.

### Extraction and analysis of total lipids

Total lipids from pericarp samples (300 mg) were extracted using the method of Wang et al.^50^. The resulting products were transesterified using 2.5% H_2_SO_4_ in methanol (v/v, 2 mL) for 1 h at 80 °C, followed by the addition of pentane (1 mL) and 0.9% NaCl (w/v, 3 mL). The pentane-containing fatty acid methyl esters in the upper phase were concentrated with a stream of N_2_, and analyzed using GC-MS with a Thermo Scientific TRACE TR-FAME GC as described by Li-Beisson et al.^10^. According to the methods for cuticular wax as described above, we identified a large amount of fatty acids with straight chain lengths ranging from C14 to C18, with zero to three double bonds, and trace amounts of VLCFAs.

### Extraction and analysis of primary metabolites

The primary metabolites were extracted with methanol (2700 μL) and 0.2 mg mL^–1^ ribitol in water (300 μL, as an internal standard) from the frozen pericarp samples (300 mg)^53^. The extracts (100 μL) were incubated in 20 mg mL^−1^ methoxyamine hydrochloride in pyridine (50 μL) for 30 min at 50 °C, and subsequently incubated in 50 μL BSTFA containing 1% TMCS for 40 min at 60 °C. Each sample was analyzed by GC-MS (Thermo Fisher, ISQII, USA) with a FID and an Agilent TR-5 MS capillary column (30 m × 25 μm i.d. × 0.1 μm). The oven, column temperature programs, and metabolite identification and annotation were as described by Yun et al.^53^.

### Extraction and analysis of secondary metabolites

The polyphenols and flavonoids were extracted with methanol (800 μL) from the dried powder of pericarp samples (100 mg)^29^. The extraction was detected using the Agilent QTOF 6520 mass spectrometer coupled to a 1200 series Rapid Resolution HPLC system (HPLC-QTOF-MS). Qualitative analysis of the metabolites followed the protocol of Ding et al.^29^.

The volatile compounds were extracted using a 15 mL solution of MTBE with 8697 mg of chlorononane and 400 mg of methyl nonanoate (as internal standards) from pericarp samples (3 g)^54^. The extract was analyzed by GC-MS (Thermo Fisher, ISQII, USA) with an FID and an Agilent DB-5 MS capillary column (30 m × 25 μm i.d. × 0.1 μm). The volatile compounds were putatively identified^53^.

### Measurement of phytohormones

ABA, SA, IAA, JA, and jasmonates were extracted from the samples (200 mg) with 800 μL of solvent containing methanol/H_2_O/acetic acid (80:19:1, v/v/v) and internal standards^52,55^. The internal standards were ^2^H_6_-ABA (Olomouc, Czech Republic) for ABA, 10-dihydro-JA (DHJA; Olomouc, Czech Republic) for JA and jasmonates, ^2^H_5_-IAA (Sigma-Aldrich, USA) for IAA, and ^2^H_6_-SA (Sigma, USA) for SA. The analysis of hormone extracts was performed using an Agilent 1100 HPLC system coupled to an Agilent API3000 mass spectrometer. Hormone identification and annotation were conducted according to Liu et al.^55^.

Three biological replicates from three fruit were sealed in a 2.5 L plastic container for 4 h and exactly 1 mL of gas was removed to quantify the levels of ethylene using an Agilent 7890 series gas chromatograph with an FID^52^.

### Metabolic network analysis

We mapped citrus genes to the AraCyc database, based on the sequence similarities with Arabidopsis, and constructed a genome-scale citrus metabolic network (CitrusCyc). To obtain CitrusCyc2.0, we removed redundant information from the CitrusCyc database, such as the sub-cellular localization data. We added information that was missing from CitrusCyc, such as the fatty acid biosynthetic pathways, based on the KEGG database (the first version) and by text mining from the literature according to Ding et al.^29^. Then, the transcriptome and metabolome data sets were mapped in CitrusCyc 2.0 by transforming the data to the parameters of the ratio value (MT vs. WT log2 fold change). The topological properties of CitrusCyc2.0 were visualized and analyzed by Cytoscape software. First, we focused on the pathways including the nodes with high degrees of connection and betweenness centrality (BC) of CitrusCyc2.0, which represented the central hub and bottlenecks in the network, respectively. Second, the correlation between the transcriptional and the metabolic nodes was analyzed in the preferential pathways of CitrusCyc2.0.

### Assays for testing resistance to fungal infection

The inoculation assay for green mould (*P*. *digitatum*), blue mould (*P. italicum*), and sour rot (*G*. *candidum*) on the surface of WT and MT fruit was used to evaluate the resistance to fungal infection with the DSI, which classifies infections into eight different levels, as described in Supplementary File 11. Pericarps from ten fruit from each variety were collected at multiple time points from 2 HPI to 3 DPI, as described by Purdy^56^. After the above-mentioned fungal infection, the decay incidence of WT and MT fruit were measured. Three biological replicates were collected from each variety at each time point.

To examine whether the wax deficiency influences the DSI and decay incidence of WT and MT fruit, epicuticular wax were removed using Arabic gum and chloroform as described by Wang et al.^49^ and Ringelmann et al.^57^. For the JA treatment, MeJA (Sigma-Aldrich) was prepared as a 100 mM stock solution with ethanol, and was adjusted to 10 μM MeJA by dilution in distilled water. ‘Lane late’ navel orange, late mature *Citrus clementina*, WT and MT fruit were dipped in 10 μM MeJA for 10 min and distilled water was used as a control as described by Droby et al.^25^. For the JA inhibitor treatment, above-mentioned fruit were dipped in 100 μM PG (Sigma-Aldrich), 100 μM IBU (Sigma-Aldrich) and 1.5 mM DIECA (Sigma-Aldrich) for 30 min, and distilled water was used as control^20–22^. The inoculation and incubation followed the same procedures described above.

### Determination of antioxidant capacity and enzymatic activities

To determine H_2_O_2_ content, 0.5 g of pericarps was extracted with 9 mL of cold 5% trichloroacetic acid, containing 0.5 g of polyvinyl polypyrrolidone (PVPP) overnight as described in Patterson et al.^58^. The extracts were centrifuged at 10,000 rpm for 20 min at 4 °C. The supernatant was neutralized to pH 7.5 with 17 M NH_4_OH, and used immediately to measure H_2_O_2_ content using a Shimadzu UV-1800 spectrometer (Shimadzu Inc., Kyoto, Japan) as described in Patterson et al.^58^.

To quantify the levels of anti-superoxide anions, peroxidase (POD), and catalase (CAT) activities, 0.5 g of pericarps were extracted with 4.5 mL of 100 mM PBS, pH 7.4, containing 0.5 g of PVPP overnight. All of these parameters were measured using a kit (Nanjing Jiancheng Bioengineering Institute) following the manufacturer’s instructions as described by Huang et al.^59^.

### Statistical analysis

PCA of metabolites was performed to assess the metabolite variation of WT and MT fruit using SIMCA-P 11.0 software (Umetrics, Umeå, Sweden). We analyzed the relationships between transcriptome and metabolome data using a Spearman correlation computed by R (version 2.14.2) software. The statistical significance of the differences between WT and MT fruit was tested using Duncan’s test (*p* < 0.05) with the SAS v8.1 software (SAS Institute, Cary, NC, USA). Mean values and standard errors bars are provided.

## Electronic supplementary material

Supplementary Figure S1-11

Supplementary File 1 Genomic SSR and EST-SSR primers

Supplementary File 2 Differentially expressed genes (DEGs) (MT vs. WT, P ≤ 0.05 and fold change ≥ 2)

Supplementary File 3 Validation of genes selected from the RNA-seq analysis and two sub-networks of CitrusCyc2.0 with quantitative real-time PCR

Supplementary File 4 Functional categorization of the genes with increased and reduced expression based on Gene Ontology (GO) annotation

Supplementary File 5 Classification of DEGs for hormone pathways

Supplementary File 6 Putatively identified metabolites based on UV-VIS spectrophotometry, GC, the untargeted GC-MS, and LC-MS

Supplementary File 7 Characteristics of CitrusCyc2.0

Supplementary File 8 Characteristics of a fine network (MT vs. WT log2 fold change: 0.3 or greater) from the overlay CitrusCyc2.0

Supplementary File 9 Amount and composition of cuticular wax in WT and MT fruits in 2013

Supplementary File 10 Characterization of the disease tolerance of the MT and WT fruits under different treatments

Supplementary File 11 Supporting experimental procedures

Supplementary File12. Primer sequences used for quantitative RT-PCR
