# Supplementary material for: Integrated transcriptomic and metabolomic analyses of a wax deficient citrus mutant exhibiting jasmonic acid-mediated defense against fungal pathogens
Source: Hortic Res. 2018 Aug 1;5:43. doi: 10.1038/s41438-018-0051-0 (PMC6068166; doi:10.1038/s41438-018-0051-0)
Supplement: Supplementary file 1 — Supplementary Figure S1-11 [file 41438_2018_51_MOESM1_ESM.doc]

**Supplementary Figures**

**Supplementary Figure S1.** Inoculation experiments with *P. digitatum*, *P. italicum* and *G. candidum* in the WT and MT fruits. (a) The rate of spore germination (Ge), and the percentage of germinated spore with germ tubes ≥ 20 μm in length (Gt) of *P. digitatum* within 24 h in WT and MT fruits. (b) Decay incidence and (c) DSI of WT and MT fruits after *P. italicum and G. candidum* infection, respectively. *: significant difference (P < 0.05, Duncan's test). Data were shown as means ± SE (n= 3).

**Supplementary Figure S2.** Score plot from the principal component analysis (PCA) of metabolites in MT and WT fruits. The top four metabolites which negatively or positively contribute to PC1 or PC2 are indicated with right triangles. Abbreviations: 26AL_wax or 28 AL_wax indicate the aldehydes with chain lengths ranging from C26 to C28.

**Supplementary Figure S3.** Metabolite contents of WT and MT fruits.

The bars represent the log2-fold change in the metabolite content ratio between WT and MT. The bars facing the right (positive) indicate a decreased content. The bars facing the left (negative) indicate an increased content in MT. Different metabolite families are indicated with different colors: red, phenolics (Phe); blue, flavnoids (Flads); green, volatile components; cyan, amine derivatives (Ad); yellow and orange, sugar and sugar alcohols (Su and Su alcohols); dark blue, organic acids (Or acids). * indicates a statistically significant difference (P < 0.05, Duncan's test). Data are presented as means ± SE (n= 3).

**Supplementary Figure S4.** General map of CitrusCyc2.0.

**Supplementary Figure S5.** Profiles of cuticular lipids and total fatty acids from the fruits of WT and MT. (a) Amount and composition of cuticular wax from WT and MT fruits. (b) Wax load of WT and MT fruits. (c) Cutin monomers from WT and MT fruits. (d) Long chain and (e) very long chain fatty acids of WT and MT fruits. *: significant difference (P < 0.05, Duncan's test). Data are presented as means ± SE (n= 3). Abbreviations: very-long-chain fatty acids, VCLFAs; long-chain fatty acids, LCFAs; 16-hydroxy-10-oxo-hexadecanoic acid, Ha; 10,16-Dihydroxy-hexadecanoic acid, Di; 16-hydroxy hexadecanoic acid, Hh; p-Hydroxy-cinnamic acid, Hc; 18-hydroxy octadeca-9-octadecenoic acid, Ho; 9,10-dihydroxyoctadecane-1.18-dioic acid, Hd; Glycerol, Gl; Octadecanoic acid, Ot.

.

**Supplementary Figure S6.** Change in the properties and longitudinal structure of WT and MT fruit surfaces.

Images of WT (a) and MT (b) fruit surfaces observed by lipid staining with Sudan IV staining and a light microscope. Scale bars: 20 μm. TEM analysis of the WT (c) and MT (d) fruit surfaces. Scale bars: 1 μm. Contact angles on the fruit surfaces of WT (e) and MT (f). (g) Weight loss of WT and MT fruits. (h) Permeability of carotenoid on the WT and MT fruit surfaces. *: significant difference (P < 0.05, Duncan's test). Data are shown as means ± SE (n= 15). Abbreviations: cuticular membrane, Cm; anticlinal peg, Ap; epidermal cell, Ec; cytoplasm, Cyt; polysaccharide cell wall, Pcw.

**Supplementary Figure S7.** Characterization of JA, SA and ethylene defense responses in WT and MT fruits after the inoculation. Abbreviations: *Endochitinase1*, *ENCH1*; *Enhanced disease susceptibility 5*, *EDS5*, *Isochorismate synthase 1*, *ICS1*; *Phenylalanine ammonialyase 1*, *PAL1*; *Azelaic acid-induced 1*, *AZI1*. *: significant difference (P < 0.05, Duncan's test). Mean values and SE bars are provided (n=3).

**Supplementary Figure S8.** Effect of *P. digitatum* infection on MeJA and JA inhibitor-treated citrus fruits. Decay incidence of the MeJA and JA inhibitors-treated WT and MT (a) and ‘Lane late’ (b) infected with *P. digitatum*. (c) DSI, (d) decay incidence and (e) phenotype of MeJA and IBU-treated late-maturing *Citrus clementina* fruits infected with *P. digitatum*. Different letters indicate significant differences among WT, MT and their corresponding treatments, respectively (P < 0.05, Duncan's test). Data are presented as means ± SE (n=3).

**Supplementary Figure S9.** H_2_O_2_ level, enzyme activity, and stress metabolite levels in WT and MT fruits.

(a) The level of H_2_O_2_. (b) anti-superoxide anion activity. (c) POD activity. (d) CAT activity. The levels of tocopherol (e) and 5-demethylnobietin (f). *: significant difference (P < 0.05, Duncan's test). Data are presented as means ± SE (n= 3). Abbreviation: fresh weight: FW.

**Supplementary Figure S10.** Contents of JA and total fatty acid from the citrus fruits with different treatments. (a) JA level of WT and MT fruits inoculated with *P. digitatum* and water as control. The relative content of WT and MT fruits at different stages compared with WT at 1 HPI. Different letters indicate statistically significant differences (P < 0.05, Duncan's test). Mean values and SE bars are provided (n=3). (b) Amount and composition of total fatty acid from fruits of Hongkong kumquat (*Fortunella hindsii* Swingle) after the metolachlor treatment. Different letters indicate statistically significant differences (P < 0.05, Duncan's test). Data are presented as means ± SD (n=2).

**Supplementary Figure S11.** Schematics of abiotic stress using the MapMan visualization platform**.**


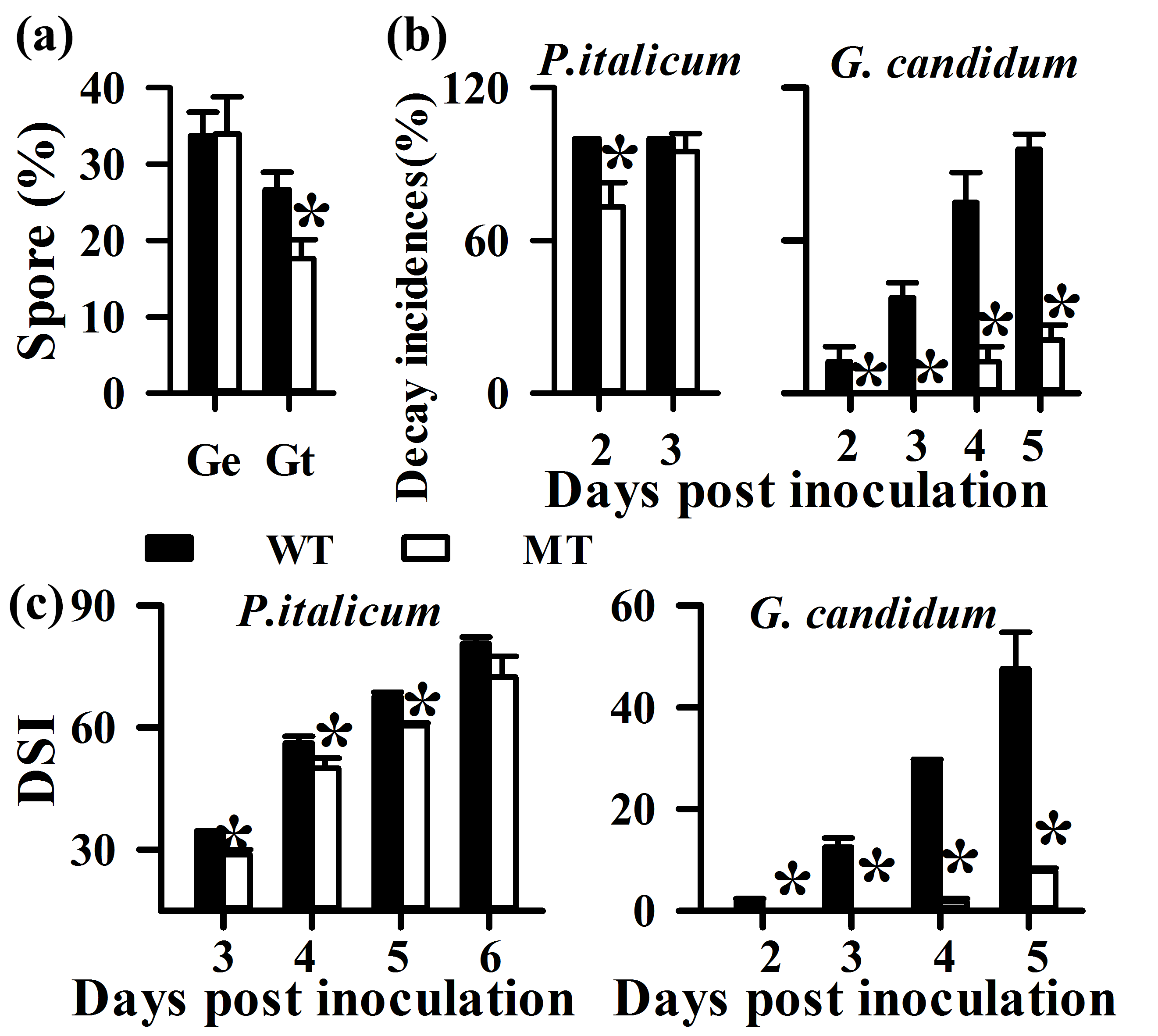


**Supplementary Figure S1**


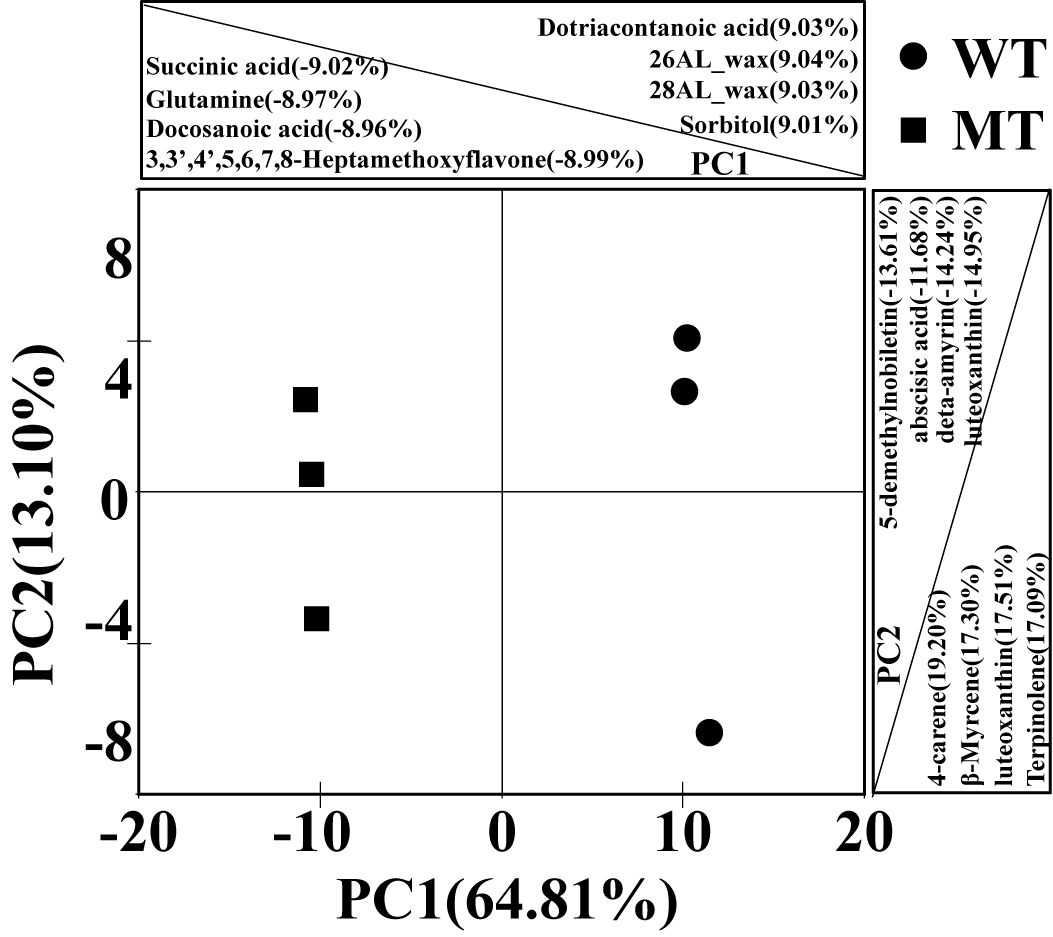


**Supplementary Figure S2**


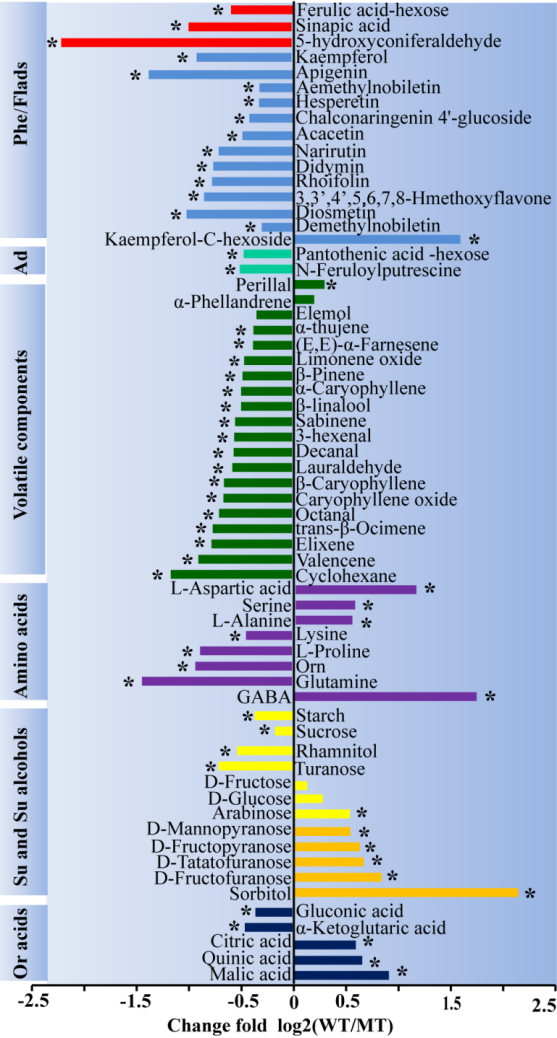


**Supplementary Figure S3**


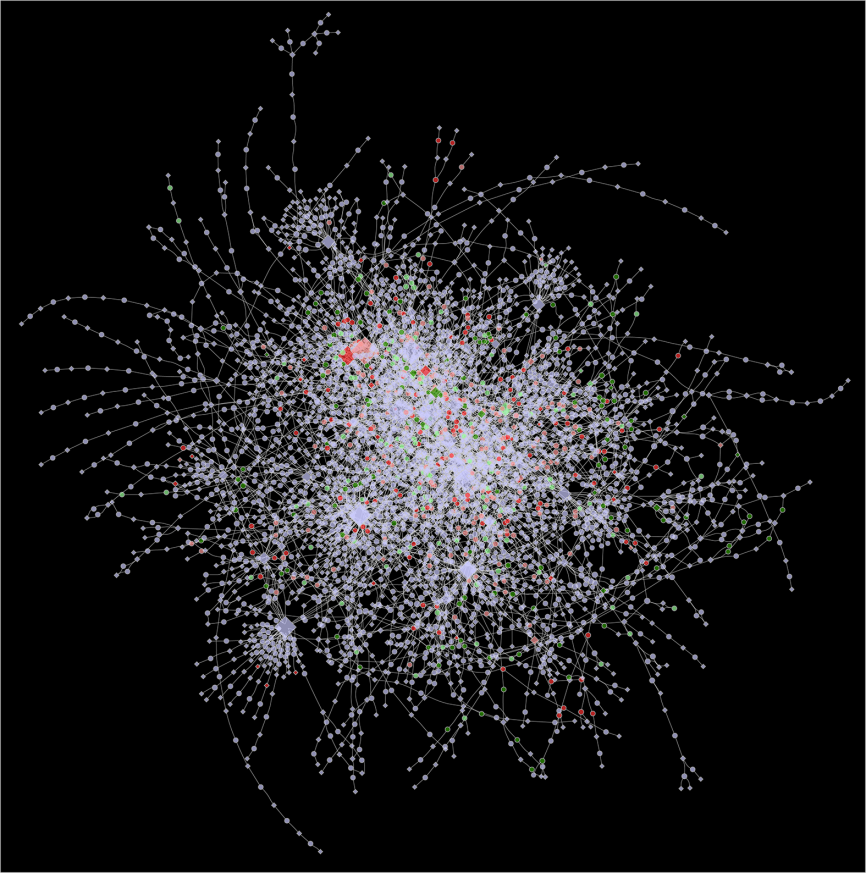


**Supplementary Figure S4**


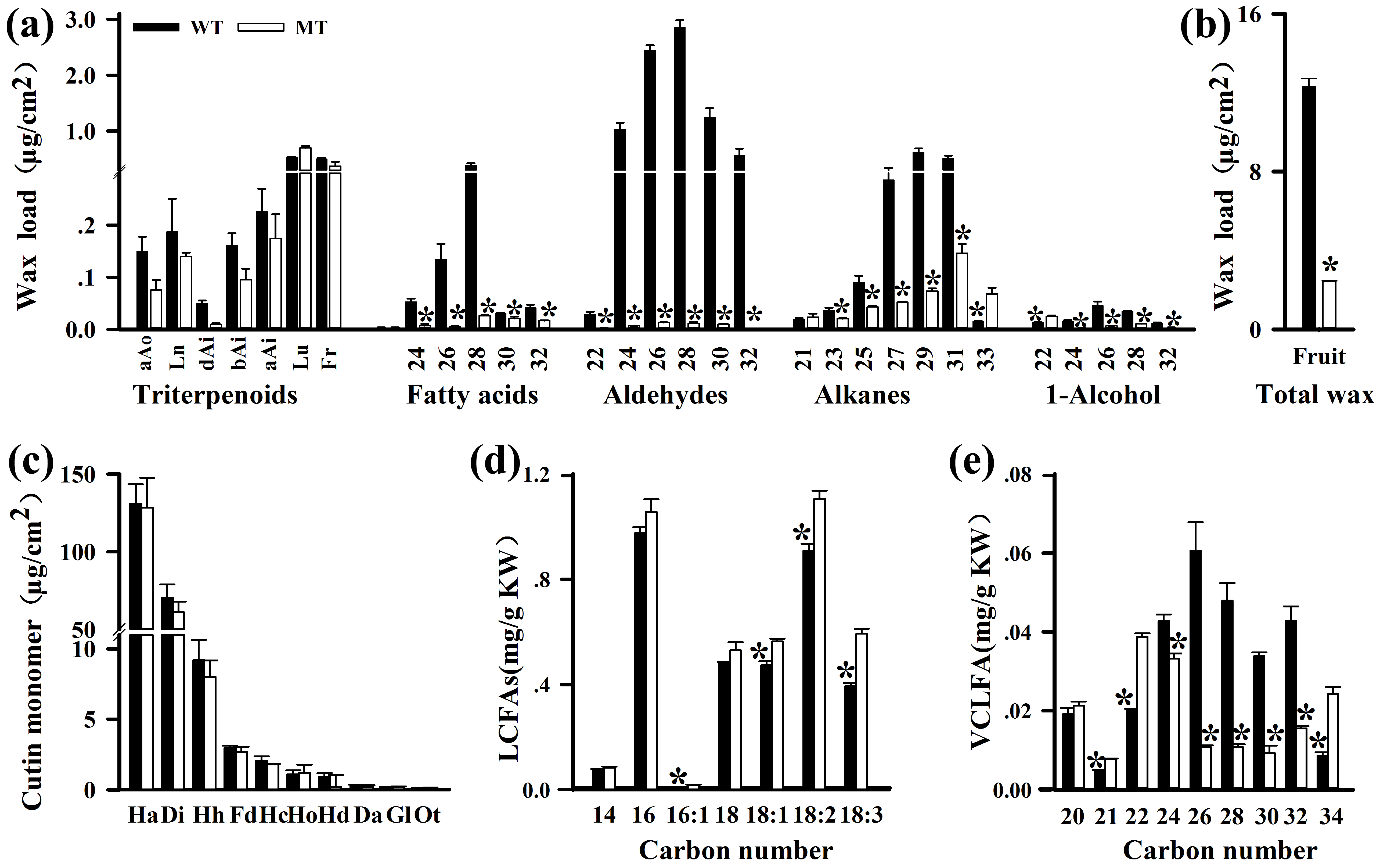


**Supplementary Figure S5**


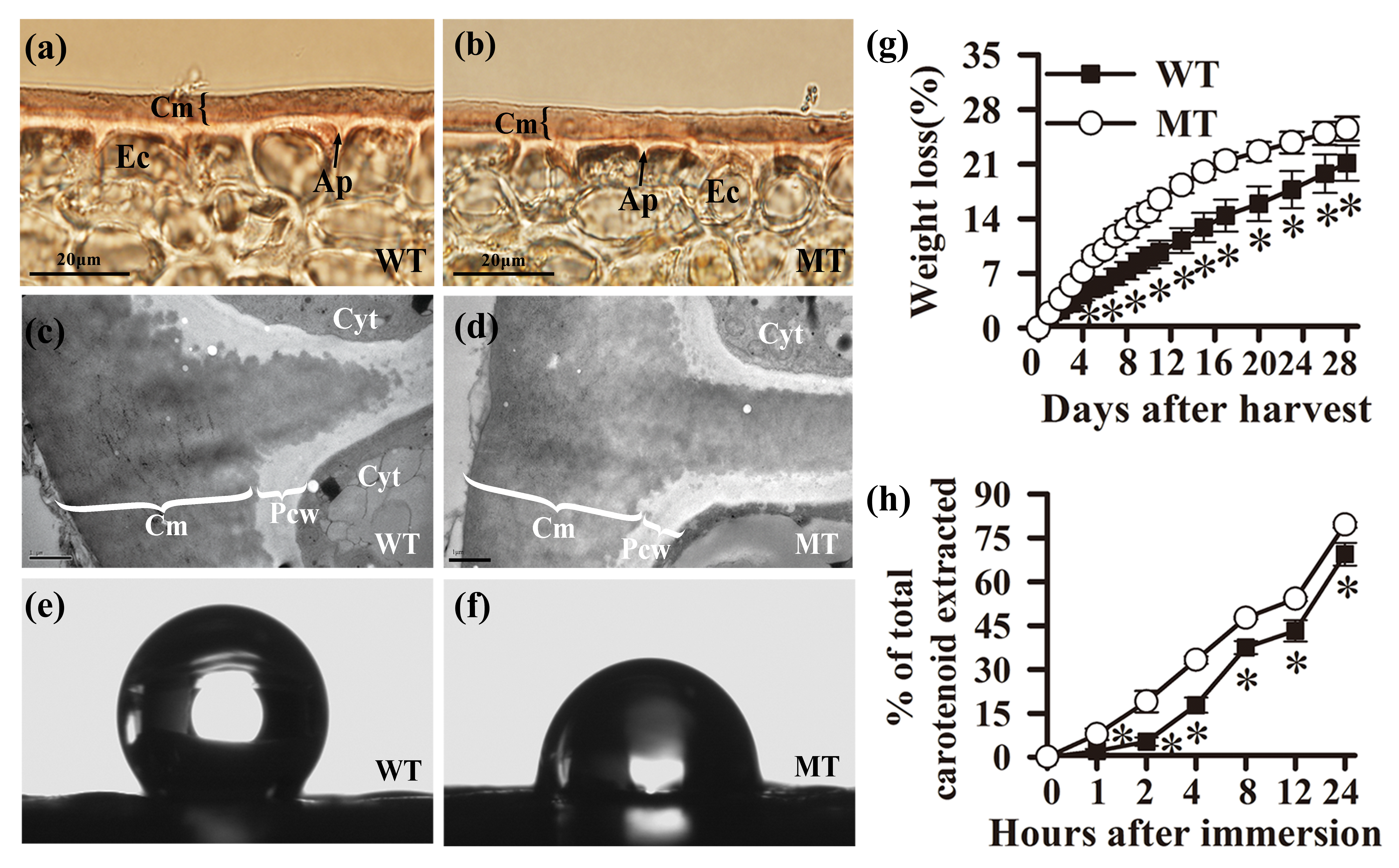


**Supplementary Figure S6**


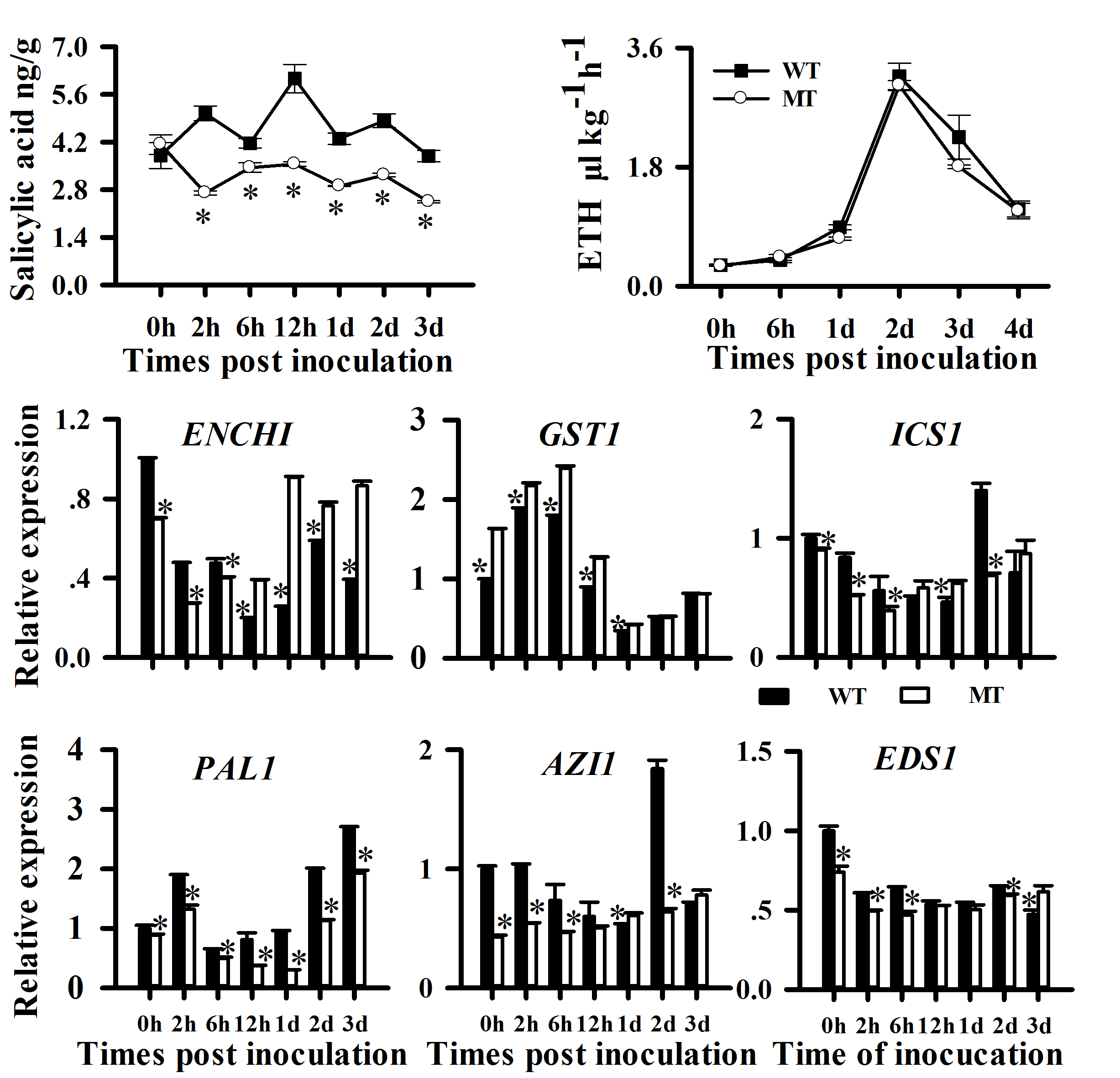


**Supplementary Figure S7**


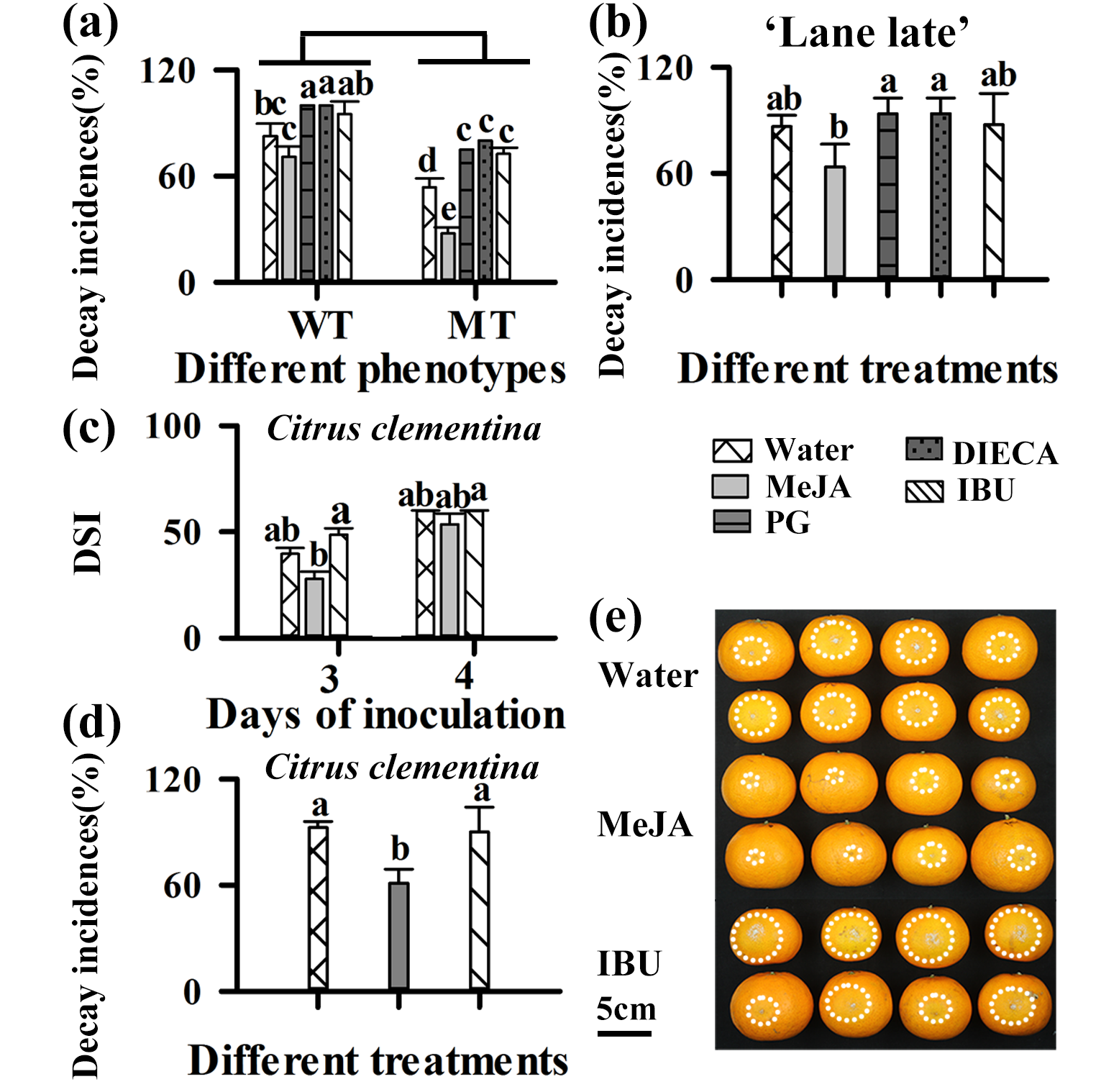


**Supplementary Figure S8**


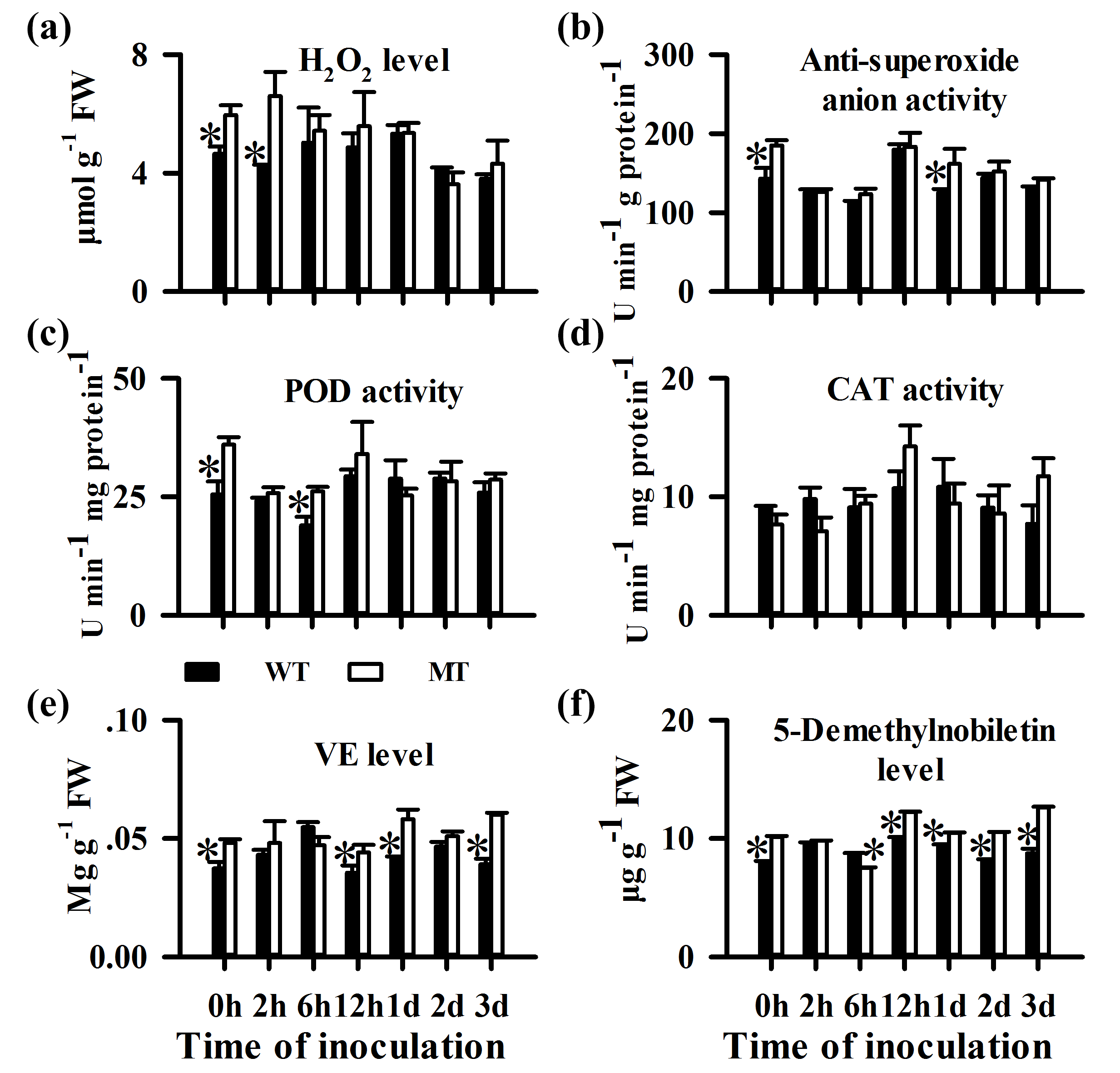


**Supplementary Figure S9**


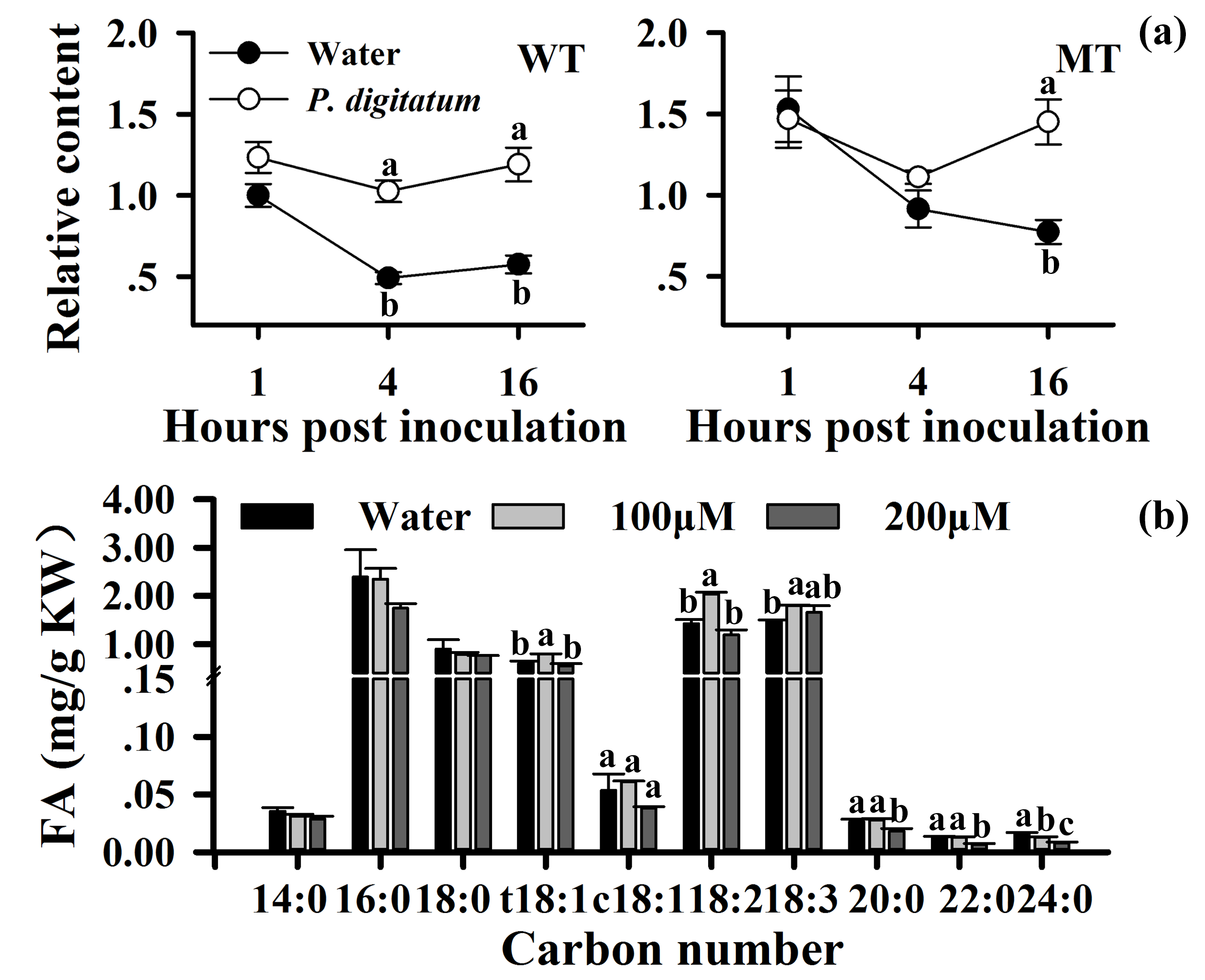


**Supplementary Figure S10**


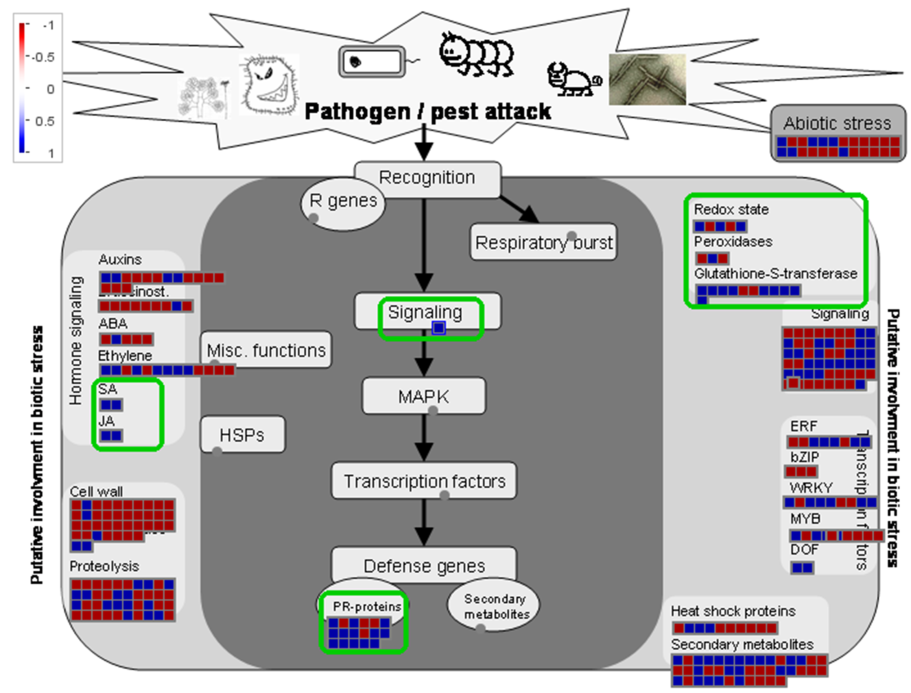


**Supplementary Figure S11**
