# Supplementary material for: Integrated transcriptomic and metabolomic analyses of a wax deficient citrus mutant exhibiting jasmonic acid-mediated defense against fungal pathogens
Source: Hortic Res. 2018 Aug 1;5:43. doi: 10.1038/s41438-018-0051-0 (PMC6068166; doi:10.1038/s41438-018-0051-0)
Supplement: Supplementary file 6 — Supplementary File 5 Classification of DEGs for hormone pathways [file 41438_2018_51_MOESM6_ESM.doc]

**Supplementary File 5.Classification of differentially Hormone-related DEGs**

| **Gene** | **TAIR-ID** | **Description** | | **E-value** | | **Regulation** |
| --- | --- | --- | --- | --- | --- | --- |
| **Abscisic acid** | | | | | | |
| ABA biosynthesis | | | | | | |
| Cs5g14370 | AT3G14440 | NCED3 | | 0 | | up |
| Cs2g03270 | AT1G30100 | NCED5 | | 0 | | down |
|  | | | | | | |
| ABA signaling | | | | | | |
| Cs8g19150 | AT1G07420 | SMO2(Sterol 4-alpha-methyl-oxidase2) | | 2.00E-138 | | down |
| Cs4g05390 | AT5G20270 | Heptahelical transmembrane protein | | 1.00E-143 | | down |
| Cs7g31880 | AT3G11410 | Phosphatase 2C | | 8.00E-151 | | down |
|  | | | | | | |
| ABA response transcription factor | | | | | | |
| orange1.1t02547 | AT2G46680 | Arabidopsis thaliana homeobox protein7 | | 5.00E-40 | | down |
| Cs5g31060 | AT3G63210 | Zinc-finger protein | | 2.00E-38 | | down |
|  | | | | | | |
| ABA responsive protein(GeneOntology) | | | | | | |
| Cs2g22010 | AT3G22060 | Receptor-like protein kinase-related family protein | | 9.00E-93 | | down |
| Cs6g09950 | AT5G59320 | lipid transfer protein 3 | | 3.00E-34 | | up |
| Cs7g02670 | AT2G38750 | Annexin-like protein RJ4 | | 3.00E-84 | | up |
|  | | | | | | |
| ABA degradation | | | | | | |
| Cs8g05290 | AT4G34131 | UDP-glucosyl transferase 73B3 | | 0 | | up |
|  | | | | | | |
| **Auxin** | | | | | | |
| Auxin biosynhesis | | | | | | |
| Cs3g07330 | AT4G31500 | | SUR2(superroot2 ) | | 2.00E-156 | down |
|  | | | | | | |
| Auxin transport | | | | | | |
| Cs6g17970 | AT1G71090 | | Auxin efflux carrier family protein | | 0 | up |
| Cs2g16620 | AT1G70940 | | Auxin efflux regulator PIN3 | | 0 | down |
|  | | | | | | |
| Auxin receptor and signaling | | | | | | |
| Cs3g14760 | AT3G05630 | [phospholipase D activity](http://amigo.geneontology.org/cgi-bin/amigo/go.cgi?action=query&view=query&query=GO:0004630&search_constraint=terms) | | 0 | | down |
|  | | | | | | |
| Auxin response transcription factor | | | | | | |
| Cs5g30390 | AT5G43700 | AUX/IAA transcriptional regulator family protein | | 3.00E-76 | | down |
|  | | | | | | |
| Auxin responsive protein | | | | | | |
| orange1.1t00546 | AT2G46690 | SAUR-like auxin-responsive protein family | | 4.00E-48 | | down |
| Cs4g18300 | AT2G26170 | MAX1(auxin polar transport) | | 0 | | down |
| Cs8g08010 | AT1G75580 | SAUR-like auxin-responsive protein family | | 1.00E-58 | | down |
| Cs3g25900 | AT1G75580 | SAUR-like auxin-responsive protein family | | 1.00E-60 | | down |
| orange1.1t04213 | AT2G21210 | SAUR-like auxin-responsive protein family | | 5.00E-35 | | down |
| Cs1g15830 | AT3G16500 | Auxin-responsive protein IAA11 | | 3.00E-44 | | down |
| Cs1g22140 | AT2G14960 | Auxin-responsive GH3 family protein | | 0 | | down |
| orange1.1t02620 | AT5G50760 | SAUR-like auxin-responsive protein family | | 2.00E-28 | | down |
|  | | | | | | |
| **Brassinosteroid** | | | | | | |
| Brassinosteroid biosynthesis | | | | | | |
| Cs3g26700 | AT1G20330 | Sterol-C24-methyltransferases | | 0 | | down |
| Cs7g31170 | AT5G05690 | C-23 steroid hydroxylase | | 0 | | down |
| Cs6g19630 | AT3G30180 | Arabidopsis CYP85A2 | | 0 | | down |
| Brassinosteroid signaling | | | | | | |
| Cs3g18070 | AT4G08950 | EXORDIUM (EXO) | | 3.00E-160 | | down |
| Cs6g21460 | AT3G29030 | Expansin A5 | | 3.00E-138 | | down |
| Cs7g11860 | AT2G26710 | Cytochrome P450 734A2 | | 7.00E-112 | | down |
| Cs7g25850 | AT5G08130 | BES1-interacting Myc-like 1 | | 2.00E-105 | | down |
| Cs7g11890 | AT2G26710 | Cytochrome P450 734A1, | | 6.00E-140 | | down |
|  | | | | | | |
| **Cytokinin** | | | | | | |
| Cytokinin biosynthesis | | | | | | |
| Cs9g06010 | AT5G19040 | Cytokinin biosynthetic process | | 9.00E-141 | | down |
|  | | | | | | |
| Cytokinin signaling | | | | | | |
| Cs3g15040 | AT2G47430 | Signal transduction histidine kinase | | 0 | | down |
| Cs2g06990 | AT4G05120 | Equilibrative nucleoside transporter | | 0 | | up |
|  | | | | | | |
| **Ethylene** | | | | | | |
| Ethylene biosynthetic | | | | | | |
| Cs3g16400 | AT4G37770 | ACS8 | | 0 | | down |
| Cs2g20590 | AT1G05010 | ACO4 | | 4.00E-128 | | up |
|  | | | | | | |
| Ethylene responsive transcription factor | | | | | | |
| Cs5g29900 | AT3G23230 | Ethylene-responsive transcription factor ERF094, | | 4.00E-37 | | up |
| Cs2g23660 | AT5G44210 | Encodes a member of the ERF (ethylene response factor) subfamily B-1 of ERF/AP2 transcription factor family (ATERF-9) | | 1.00E-52 | | down |
| Cs4g17960 | AT2G25490 | EIN3 Binding F-box protein | | 0 | | up |
|  | | | | | | |
| **Gibberellin** | | | | | | |
| Gibberellin response transcription factor | | | | | | |
| Cs9g04500 | AT5G24860 | FPF1 (flowering promoting factor 1) | | 7.00E-44 | | down |
| orange1.1t00521 | AT3G61850 | Zinc finger transcription factor of the Dof family involved in the control of seed germination. | | 1.00E-72 | | up |
| Gibberellin responsive protein | | | | | | |
| Cs6g17190 | AT1G74670 | Gibberellin-regulated family protein | | 5.00E-36 | | down |
| Cs1g24810 | AT5G66350 | Lateral root primordium (LRP) protein-related | | 1.00E-92 | | down |
| Cs8g01150 | AT4G35390 | AT-hook protein of GA feedback 1 | | 4.00E-68 | | down |
| Cs7g03630 | AT1G69530 | Expansin A1 | | 3.00E-150 | | down |
| Cs8g04550 | AT2G14900 | Gibberellin-regulated family protein | | 2.00E-27 | | down |
| Cs6g20220 | AT5G14920 | Gibberellin-regulated family protein | | 5.00E-28 | | down |
| Gibberellin biosynthetic | | | | | | |
| orange1.1t00272 | AT5G51810 | Putative gibberellin 20 oxidase | | 6.00E-171 | | down |
| Cs2g07550 | AT4G21200 | Putative gibberellin 2-oxidase | | 1.00E-161 | | down |
| Cs5g14480 | AT1G78440 | Arabidopsis thaliana gibberellin 2-oxidase 1 | | 2.00E-139 | | up |
|  | | | | | | |
| **Jasmonic acid** | | | | | | |
| Jasmonic acid biosynthetic process | | | | | | |
| orange1.1t03729 | AT1G76690 | 12-oxophytodienoate reductase 2 | | 0 | | up |
| orange1.1t03769 | AT3G45140.1 | Encodes a chloroplast lipoxygenase required for wound-induced jasmonic acid accumulation in Arabidopsis. | | 0 | | up |
|  | | | | | | |
| Jasmonic acid responsiive protein | | | | | | |
| Cs7g05210 | AT2G26690 | Major facilitator superfamily protein | |  | | up |
|  | | | | | | |
| Jasmonic acid response transcription factor | | | | | | |
| Cs6g10120 | AT3G56400 | WRKY DNA-binding protein 70 | | 5.00E-40 | | up |
|  | | | | | | |
| Jasmonic acid signaling process | | | | | | |
| Cs7g02820 | AT5G13220 | Jasmonate-zim-domain protein 10 | | 7.00E-53 | | up |
|  | | | | | | |
| **Salicylic acid** | | | | | | |
| Salicy acid response transcription factor | | | | | | |
| Cs2g12700 | AT1G68320 | MYB domain protein 62 | | 2.00E-94 | | down |
| orange1.1t02759 | orange1.1t02759 | WRKY transcription factor 18, | | 1.00E-99 | | up |
| Cs7g06330 | AT1G80840 | WRKY DNA-binding protein 40 | | 6.00E-37 | | up |
|  |  |  | |  | |  |
| Salicy acid signaling process | | | | | | |
| Cs1g20710 | AT5G26780 | Encodes a protein with serine hydroxymethyltransferase activity | | 0 | | down |
| Salicy acid responsive protein | | | | | | |
| Cs8g19400 | AT2G29420 | Glutathione S-transferase tau 7 | | 9.00E-91 | | down |
| Cs7g15760 | AT2G29420 | Glutathione S-transferase tau 7 | | 1.00E-67 | | up |
| orange1.1t03618 | AT2G29420 | Glutathione S-transferase U17, | | 4.00E-60 | | up |
| orange1.1t03617 | AT2G29420 | Glutathione transferase GST 23, | | 5.00E-60 | | up |
| orange1.1t03632 | AT2G29420 | Glutathione transferase GST 23, | | 1.00E-59 | | up |
| orange1.1t03629 | AT2G29420 | Probable glutathione S-transferase, | | 8.00E-58 | | up |
|  | | | | | | |
| Salicy acid synthesis | | | | | | |
| Cs5g21220 | AT2G43820 | UDP-glucosyltransferase 74F2 | | 1.00E-114 | | up |
| Cs1g24440 | AT3G11480 | Encoding a protein with BAMT and SAMT activities | | 4.00E-102 | | up |
