# Supplementary material for: Integrated transcriptomic and metabolomic analyses of a wax deficient citrus mutant exhibiting jasmonic acid-mediated defense against fungal pathogens
Source: Hortic Res. 2018 Aug 1;5:43. doi: 10.1038/s41438-018-0051-0 (PMC6068166; doi:10.1038/s41438-018-0051-0)
Supplement: Supplementary file 12 — Supplementary File 11 Supporting experimental procedures [file 41438_2018_51_MOESM12_ESM.docx]

**Supplementary File 11：Supplementary methods**

| **Methods name** | experimental procedures in detailed |
| --- | --- |
| **Permeability of the**  **cuticle to total**  **carotenoids of**  **mature orange fruits** | For the carotenoids leaching assay, one commercial mature fruit per phenotype was immersed in 250 mL volumes of ethanol in dark condition. The solution was measured at 450 nm to calculate the amount of total carotenoid leached by a spectrophotometer (SHIMADZU, Japan). Total carotenoid was quantified as β-carotene using a standard curve that is y=6.8422x (R^2^=0.99) as described by Lee *et al*. (2001). Three biological replicates were analyzed for each phenotype. |
| **Surface**  **hydrophobicity measurement** | Leaves and peels of fruits were fixed to glass slides with double-sided tape and 10 mL droplets of distilled water were dropped using a micropipette. The droplets was taken the photographs and the contact angle was measured using the angle tool incorporated in the image J version 1.44p software according to Uppalapati *et al*. (2012). 8 fruits per a phenotype were used to determine the contact angles. Three biological replicates were analyzed for each phenotype. |
| **Classification of**  **differentially regulated**  **putative transcription**  **factors** | To assign the putative transcription factors to the phenotype differences between the WT and MT, we compared our datasets with a total of 2256 citrus transcription factors in the PlantTFDB database (http://planttfdb.cbi.pku.edu.cn) (Jin *et al*., 2013) using the BLASTN software (E-value < 1E-10, identity ≥ 80%). The P-value of a hypergeometric distribution (P < 0.05) was calculated based on the frequency of a family with the up- or down-regulated gene sets compared with its frequency within the whole set in the transcriptional database. |
| **Assays for testing**  **resistance to**  **fungal infection** | Green mould (*P. digitatum*) and sour rot (*G. candidum*) was evaluated the resistance to fungal infections in treated fruit according to method of Yun *et al*. (2013). Two uniform lesions (5 mm in diameter, 3 mm in depth) were created at the equator of 120 sterilized fruits for WT and MT, respectively. Aliquots of 20 μL containing a 1.0 × 10^6^ spore mL^-1^ conidial suspension were inoculated at the wound sites. Flavedos were collected from 2 h to 3 d time post inoculation (TPI). The resistance was rated with three replicates according to the disease severity index (DSI) as described by Purdy (1979), with minor modifications, which was classified into seven levels: 0 (no lesion ≤ 0.5 cm2), 1 (lesion area ≤ 1 cm^2^), 2 (lesion area ≤ 2 cm^2^), 3 (lesion area ≤ 4 cm^2^), 4 (lesion area ≤ 12 m^2^), 5 (lesion area ≤ 20 cm^2^), 6 (lesion area ≤ 40 cm^2^) and 7 (lesion area > 40 cm^2^). The lesion diameters were measured to calculate the lesion areas. The DSI was calculated with the following formula: DSI = Σ (class × number of fruits in class) × 100/ (total number of fruits × 7), where 7 is the maximum disease rating value. |
|  |  |

**References**

**Lee H S, Castle W S, Coates G A.** 2001. High-performance liquid chromatography for the characterization of carotenoids in the new sweet orange (Earlygold) grown in Florida, USA. *Journal of Chromatography A*, 913(1): 371-377.

**Jin J, Zhang H, Kong L, Gao G, Luo J.** 2013. PlantTFDB 3.0: a portal for the functional and evolutionary study of plant transcription factors. *Nucleic Acids* Res, gkt1016.

**Uppalapati SR, Ishiga Y, Doraiswamy V, Bedair M, Mittal S, Chen J, Nakashima J, Tang Y, Tadege M, Ratet P. 2012.** Loss of abaxial leaf epicuticular wax in Medicago truncatula irg1/palm1 mutants results in reduced spore differentiation of anthracnose and nonhost rust pathogens. Plant Cell 24, 353-370.

**Purdy L.H.** 1979. Sclerotinia sclerotiorum: history, diseases and symptomatology, host range, geographic distribution, and impact. *Phytopathology* 69, 875-880.

**Yun Z, Gao H, Liu P, Liu S, Luo T, Jin S, Xu Q, Xu J, Cheng Y, Deng X.** 2013. Comparative proteomic and metabolomic profiling of citrus fruit with enhancement of disease resistance by postharvest heat treatment. *BMC plant biology* 13, 44.
